# Omega-3 fatty acids and major depression: a Mendelian randomization study

**DOI:** 10.1038/s41398-024-02932-w

**Published:** 2024-05-29

**Authors:** R. Carnegie, M. C. Borges, H. J. Jones, J. Zheng, P. Haycock, J. Evans, R. M. Martin

**Affiliations:** 1 Centre for Academic Mental Health, Population Health Sciences, Bristol Medical School, University of Bristol, Bristol, UK; 2 Medical Research Centre (MRC) Integrative Epidemiology Unit (IEU), University of Bristol, Bristol, UK; 3 Population Health Sciences, Bristol Medical School, University of Bristol, Bristol, UK; 4 NIHR Biomedical Research Centre at University Hospitals Bristol and Weston NHS Foundation Trust and the University of Bristol, Bristol, UK

**Keywords:** Depression, Genomics

## Abstract

Omega-3 fatty acids have been implicated in the aetiology of depressive disorders, though trials supplementing omega-3 to prevent major depressive disorder (MDD) have so far been unsuccessful. Whether this association is causal remains unclear. We used two sample Mendelian randomization (MR) to investigate causality. Genetic variants associated with circulating omega-3 and omega-6 fatty acids in UK Biobank (UKBB, *n* = 115,078) were selected as exposures. The Psychiatric Genomics Consortium (PGC) genome-wide association studies (GWAS) of MDD (*n* = 430,775; cases = 116,209; controls = 314,566) and recurrent depression (rMDD, *n* = 80,933; cases = 17,451; controls = 62,482), were used as outcomes. Multivariable MR (MVMR) models were used to account for biologically correlated lipids, such as high- and low-density cholesterol and triglycerides, and to explore the relative importance of longer-chain omega-3 fatty acids eicosapentaenoic acid (EPA) and docosahexaenoic acid (DHA) using data from the Cohorts for Heart and Aging Research in Genomic Epidemiology (CHARGE, *n* = 8866). Genetic colocalization analyses were used to explore the presence of a shared underlying causal variant between traits. Genetically predicted total omega-3 fatty acids reduced the odds of MDD (OR_IVW_ 0.96 per standard deviation (SD, i.e. 0.22 mmol/l) (95% CIs 0.93–0.98, *p* = 0.003)). The largest point estimates were observed for eicosapentaenoic acid (EPA), a long-chain omega-3 fatty acid (OR_EPA_ 0.92; 95% CI 0.88–0.96; *p* = 0.0002). The effect of omega-3 fatty acids was robust to MVMR models accounting for biologically correlated lipids. ‘Leave-one-out’ analyses highlighted the *FADS* gene cluster as a key driver of the effect. Colocalization analyses suggested a shared causal variant using the primary outcome sample, but genomic confounding could not be fully excluded. This study supports a role for omega-3 fatty acids, particularly EPA, in the aetiology of depression, although pleiotropic mechanisms cannot be ruled out. The findings support guidelines highlighting the importance of EPA dose and ratio for MDD and question whether targeted interventions may be superior to universal prevention trials, as modest effect sizes will limit statistical power.

## Introduction

**Fig. 1:**
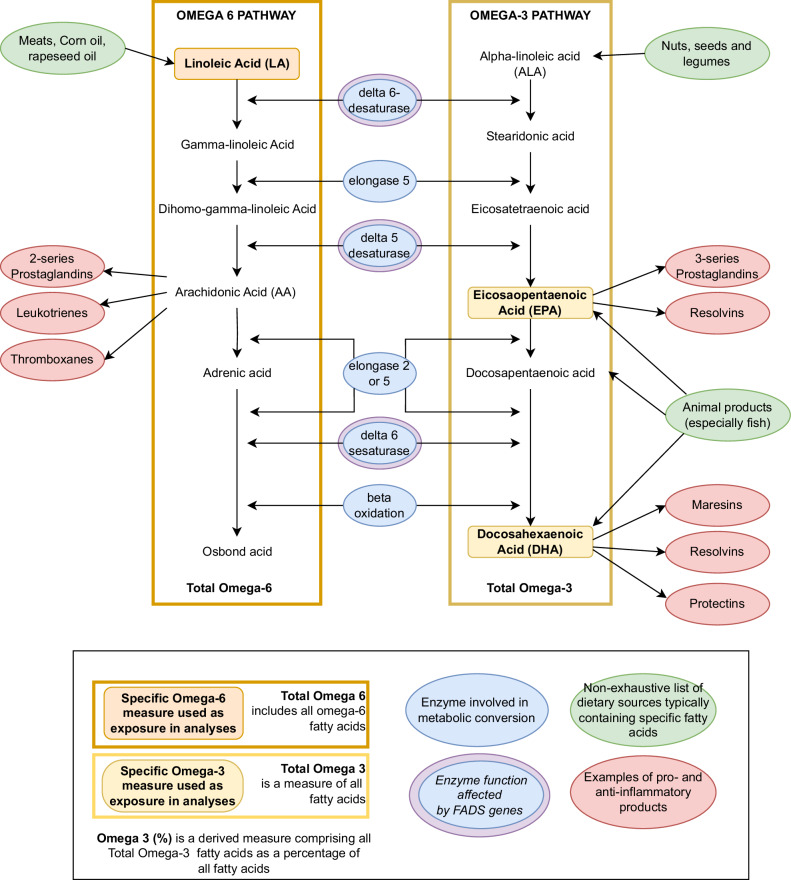
Overview of omega-3 and omega-6 fatty acid metabolism. Omega 3- and -6 fatty acid metabolic pathway, including some common dietary sources, shared enzymes involved in desaturation and elongation, and some derived products of inflammatory importance. Specific Omega-6 and Omega-3 fatty acids included as exposures in MR analyses (i.e. EPA, DHA, and LA) are highlighted in a biological context. Total Omega-3 and -6 fatty acid measurements include measures of all the fatty acids of these subtypes. Omega-3% is a further derived measurement of the percentage of Omega-3 fatty acids to total fatty acids. The pathway, products and food sources are oversimplified for clarity. Although many of the omega-6 fatty acid AA derivatives are pro-inflammatory, and more of the omega-3 fatty acid EPA and DHA derivatives are considered lower inflammatory or anti-inflammatory, this is also an oversimplification.

Omega-3 fatty acids are essential micronutrients found throughout the diet and incorporated into cell membranes across the body [1]. Long-chain omega-3 fatty acids, such as eicosapentaenoic acid (EPA) and docosahexaenoic acid (DHA), are particularly important for brain health, with direct effects on neuronal membrane fluidity, neurotransmitter release, and protection against apoptosis and cell death [2, 3]. Omega-3 fatty acid intake also moderates systemic inflammatory responses, partly by competitive inhibition of pro-inflammatory omega-6 fatty acid derivatives [1]. The most efficient dietary source of long-chain omega-3 fatty acids EPA and DHA is the consumption of oily fish, a high intake of which has been associated with numerous health benefits [4, 5]. Shorter-chain omega-3 fatty acids, such as alpha-linoleic acid (ALA) contained in nuts and seeds, can be endogenously converted into long-chain omega-3 fatty acids using a series of elongation and desaturation reactions, which are depicted in Fig. 1 [6]. However, these processes are highly variable and relatively inefficient, with other fatty acids competing for metabolic pathways. Increased omega-6 fatty acid consumption from seed oils such as corn, soybean, and safflower oil may reduce endogenous longer-chain omega-3 fatty acid production through competition of metabolic pathways [6]. High omega-6:3 fatty acid ratios- a feature of typical Western diets- have been associated with systemic inflammation and poor health outcomes, including major depression [7, 8].

Major depressive disorder (MDD) is a debilitating mental condition characterized by pervasive feelings of sadness and loss of interest and often accompanied by feelings of guilt, hopelessness, low energy and concentration, disturbances in sleep, appetite and suicidal ideation [9]. Despite progress in treatment options for MDD, it remains a leading cause of global morbidity, affecting individuals, families, and societies [9, 10]. Each episode of MDD increases the likelihood of recurrence, with a significant proportion of individuals experiencing a chronic course of relapse and remission. Given the high prevalence, recurrence, and impact of MDD, interventions with even modest preventative effects may have significant population impacts.

The association between omega-3 fatty acids and MDD has been a focus of research for decades, but uncertainty about its true nature remains. While observational studies suggest inverse associations between omega-3 fatty acids and MDD [7, 11], estimates are likely to be confounded by broader dietary consumption, socioeconomic factors [5], as well as reverse effects from poorer dietary habits among depressed individuals [12]. Trials using omega-3 fatty acid supplements for MDD have yielded mixed results [12–15], possibly due to methodological heterogeneity [16]. Recent meta-analyses have highlighted the impact of dose and EPA:DHA ratio on MDD outcomes [17, 18] with a minimum of 1–2 g per day and a minimum ratio of 2:1 advocated in current guidelines [16]. Differences in selected populations, background dietary intakes, omega-3 measurements, MDD categorization, and oxidation of omega-3 fatty acid supplements over time [19] may also complicate the planning and interpretation of trials. Trials using omega-3 fatty acids to prevent MDD could provide stronger evidence for an aetiological role, although these are more practical and financially onerous [13, 14]. Two recent prevention trials have not identified any benefit of omega-3 fatty acids above placebo on MDD incidence [12, 15]. In fact, the VITAL-DEP trial identified an increased risk of MDD from 1 g fish oil per day over 5 years (HR 1.13 (1.01–1.26), *p* = 0.03, *n* = 9171) [15], although the authors acknowledged the EPA dose (465 g) and ratio (<2:1) as a limitation. Similarly, the omega-3 arm of the MooDFOOD trial found no significant improvement of 1412 mg of EPA and DHA (3:1 ratio) on depressive symptoms in 682 participants over a 12-month follow-up compared to placebo [12]. However, baseline MDD scores were high, and regression to the mean across groups limited statistical power. Furthermore, the MooDFOOD supplements included a combination of micronutrients, differential effects of which may have impacted outcomes. It is hard to ascertain whether minor methodological adaptations may have affected trial outcomes, and further investigation of causality may help to inform successful intervention development.

Mendelian randomization (MR) is an epidemiological method that can be used to investigate causality with fewer risks and financial costs than large-scale interventional research. MR uses genetic variants as ‘instrumental variables’ for exposures, to reduce the impact of confounding and reverse causation [20, 21]. Two previous MR studies have investigated omega-3 fatty acids in MDD [22, 23]. Although neither study identified strong evidence for a causal effect, they were unable to exclude a clinically important effect due to the limited availability of genetic data. The first MR used Avon Longitudinal Study of Parents and Children data to investigate the MR association between maternal DHA and perinatal depression (RD −0.09 (−0.23 to 0.05) *p* = 0.20, *n* = 2378). [22] The second used seven genetic variants relating to omega-3 to investigate the association with MDD in the Psychiatric Genomics Consortium (PGC) MDD sample (OR 0.94; 95% CI 0.87–1.17; *p* = 0.16, *n* = 480,359)), and in the Netherlands Study of Depression and Anxiety cohort (OR 1.01, 95% CIs 0.90–1.14, *p* = 0.83, *n* = 2047) [23].

The latest Genome-Wide Association Study (GWAS) of omega-3 and -6 fatty acids in UK Biobank (UKBB) [24] includes nearly five times the sample size of previous studies, increasing the power to detect modest but potentially important effects [24]. We, therefore, undertook a comprehensive two-sample MR study of multiple omega fatty acids in MDD [25] and recurrent depression (rMDD) [26] to explore whether genetically predicted omega-3 fatty acids reduce MDD risk and to explore whether long-chain omega-3 fatty acids EPA and DHA have differential effects, which might partly explain variability among intervention trial outcomes.

## Methods

**Fig. 2:**
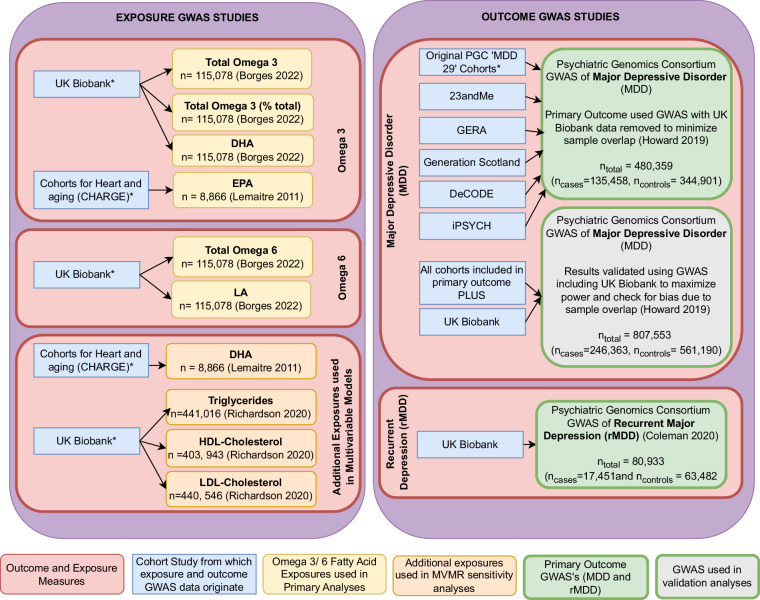
An overview of GWAS datasets used for exposures and outcomes. Further information on the GWAS datasets, including access, is given in supplementary material (S2). As our main exposure GWASs were from UK Biobank, our primary outcome sample was a GWAS with UK Biobank participant data excluded. The complete MDD GWAS sample was used for validation, to check for bias due to sample overlap, and to maximize analytical power (results provided in supplementary material, S5). GWAS summary statistics for the majority of studies are freely available, see supplementary material.

**Fig. 3:**
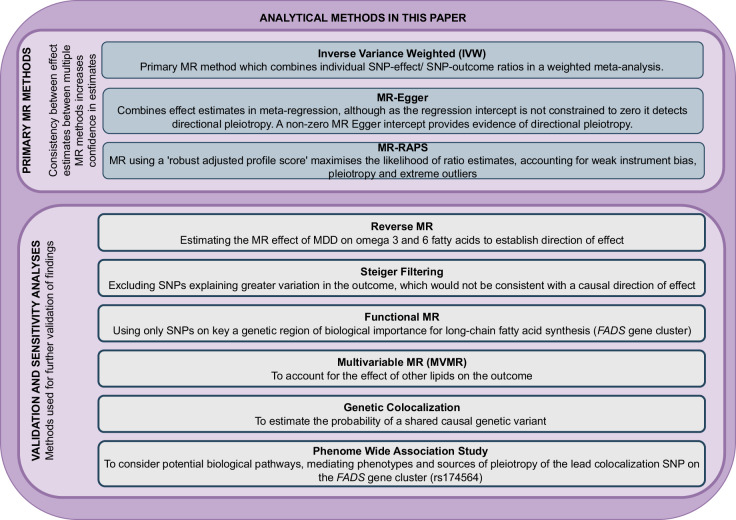
Overview of MR methods. An overview of MR methods used in this paper, with an explanation of rationale. Consistency of MR effect estimates between methods increases the strength of findings.

An overview of data and methods used in this study are summarized in Figs. 2 and 3.

We undertook two-sample MR analyses using summary-level genetic data derived from existing GWAS studies (Fig. 2*)*. We used genetic variants (single nucleotide polymorphisms (SNPs)) identified as robustly associated with fatty acids exposures in UKBB [24], with SNP-outcome associations taken from PGC MDD [25] and rMDD [26] GWASs. We estimated the effect of genetically predicted increased fatty acid exposures on the odds of MDD and rMDD, looking for consistency between MR estimates. Figure 3 details MR methods and further validation analyses used to confirm the effect direction, biological plausibility, and possible alternative pathways.

### Data sources

#### Genetic association data for fatty acids

SNPs were selected from GWASs of circulating measures of omega fatty acids from 115,078 participants of European ancestry in the UK Biobank (UKBB) [24] (see Fig. 2). Fatty acids were measured using a targeted high-throughput nuclear magnetic resonance (NMR) platform (Nightingale Health Ltd), which measures 165 metabolic measures (and a further 84 derived ratios) and normalized using inverse rank-based normal transformation [24]. Omega-3 fatty acid exposures included total omega-3 fatty acids (mmol/L), percentage of omega-3 to total fatty acids (%) and DHA. Total omega-6 and its longer-chain derivative linoleic acid (LA) were used as comparators. One standard deviation (SD) of each exposure corresponded to a change of ~0.22 mmol/l of total omega-3, 0.08 mmol/l for DHA, 1.56% for omega-3 (%), 4.45 mmol/L of total omega-6 and 3.41 mmol/L of LA, although precise quantification may be affected by normalization processes prior to GWAS.

As EPA was not measured on the NMR platform in UKBB, we obtained SNP- exposure effect sizes from Cohorts for Heart and Aging Research in Genomic Epidemiology (CHARGE) Consortium fatty acid GWAS’s (*n* = 8866), for EPA analyses, and in multivariable models with DHA. SNP-exposure effect sizes were converted to mmol/L and standardized to improve comparability with the UKBB GWAS (more details in S1).

#### Genetic association data for depression

Outcome data were obtained with permission from the PGC MDD working group. The PGC MDD sample [25] contains genotype data from 807,533 participants. Studies contributing data to the PGC MDD GWAS are shown in Fig. 2, with inclusion, exclusion and diagnostic criteria detailed in supplementary Table S2. The MDD GWAS combined studies with clinically defined diagnostic criteria (referred to as PGC_139k) [27] with genetic data from 23andme and UKBB, defining cases via self-report. Despite diagnostic variability, a strong genetic correlation exists between the clinically defined PGC_139K sample and 23andme (*r*_G_ = 0.85 (se = 0.03)) and UKBB samples (*r*_G_ = 0.87 (se = 0.04). To avoid potential bias induced by sample overlap with the exposure GWAS, we used summary statistics with UKBB data removed for primary analyses, leaving 135,458 MDD cases and 344,901 controls. We compared our findings to analyses using the complete PGC cohort (including UKBB participants) to confirm results and maximize power (246,363 cases and 561,190 controls). This also enabled us to observe whether our findings were affected by sample overlap, which was unavoidable for our secondary outcome measure (rMDD). Summary statistics for rMDD were obtained from a PGC subsample based on DSM-5 diagnoses from an online mental health questionnaire in UKBB [26]. This GWAS classified rMDD cases as individuals reporting multiple depressive episodes across their lifetime (*n* = 17,451) and controls as those with no prior episodes of MDD (*n* = 63,482) [26].

### Selection of fatty acid instruments

SNPs were selected as potential genetic instruments if they reached traditional genome-wide p-value thresholds in the relevant GWAS (*p* < 5 × 10^−8^). SNP sets were clumped to remove those in high linkage disequilibrium (threshold *r*^2^ < 0.001), using the 1000 genomes European LD reference panel. SNP-exposure effect estimates were standardised to reflect a standard deviation change in each exposure. Full details of methods and SNP sets for all exposures are provided in supplementary material (S1 and S3).

### Statistical analysis

Analyses were undertaken in R 4.0.2 [28]. Genetic instruments were identified using the ieugwasr package (version 0.1.5), using the publicly available GWAS data from the IEU Open GWAS project [29]. The computer code used to undertake MR analyses is published in an online repository (see https://github.com/eprec/Omega3_MDD).

The TwoSampleMR analysis package (version 0.5.6) [30] was used to harmonize the SNP-exposure and SNP-outcome data and derive odds ratio (OR) estimates for each SNP (Wald ratios) per SD change in the exposure. We used three MR methods to estimate the effect of genetically predicted fatty acids on the odds of MDD and rMDD, as each method has its own limitations and assumptions. The primary MR method, Inverse Variance Weighted (IVW) analysis, regresses the SNP-outcome on the SNP-exposure effect estimates, forcing the intercept to be zero and using inverse variance weights. As IVW estimates can be biased by the presence of horizontal pleiotropy (where the genetic variant influences the outcome by a mechanism other than through the exposure), we also used MR-Egger, which relaxes the zero intercept for analyses, highlighting unbalanced pleiotropy through the MR-Egger intercept. Our third MR method, MR-RAPS (Robust Adjusted Profile Score), can give a robust MR estimate in the presence of many weak instruments. Cochran’s and Rucker’s Q were calculated to investigate heterogeneity across instruments in each analysis.

MR sensitivity plots for each exposure and a forest plot combining all fatty acid OR_IVW_ estimates were created using the ggplot2 R package [31]. Sensitivity plots for each MR analysis included a scatter plot to compare MR estimates between methods, a funnel plot of MR instrument precision, forest plots of individual SNP Wald ratios, and ‘leave-one-out’ analyses showing IVW estimates after omitting each SNP. Consistent estimates across MR methods and sensitivity analyses increase confidence in the validity of the findings.

### Validation of findings

As many of the SNPs identified in the omega-3 fatty acid GWAS map to genes that are either of unknown relevance to omega-3 fatty acid metabolism or thought to be related to lipoprotein metabolism more broadly, we undertook several supplementary analyses to investigate consistency, biological mechanisms, and potential pleiotropy, which are summarized in Fig. 3 and detailed in supplementary methods S1. Statistical power for these analyses was variable, so the primary aim was to check consistency in the magnitude of effect across results.

Firstly, we undertook reverse MR and Steiger Filtering [32] to investigate the direction of the effect. Secondly, we restricted analyses to a key biologically plausible pathway, using an SNP on the *FADS* gene cluster, known to relate to omega fatty acid desaturation (see Fig. 1 for further context) [33]. Thirdly, we used multivariable MR (MVMR) [34] to investigate whether the effects could be driven by pleiotropic effects on genetically correlated lipids and to explore the comparative effects of longer chain fatty acids- EPA and DHA- on MDD. The number of exposures in MVMR models was limited by conditional *F* statistics (i.e. the strength of instruments conditioned on the other exposures [35]), which were reduced with additional exposures. In addition to affecting the precision of our estimates, weak instruments in MVMR can lead to bias in unpredictable directions [35].

We used genetic colocalization [36] to estimate the probability of a shared causal variant in the *FADS* region underlying variation in both circulating omega-3 and MDD. Finally, we undertook a phenome-wide association study (PheWAS) of the lead *FADS* SNP driving the apparent causal effect on MDD (*rs174564)* to consider potential biological pathways, mediating phenotypes and sources of pleiotropy downstream of that SNP.

## Results

### Genetic instruments

Genetic instruments for fatty acid exposures explained between 3.2% and 9.3% of the variance of each exposure (see supplementary material S2). Mean F-statistics were 120- 8572 for UKBB fatty acid exposures and 15–6315 for *FADS* analyses, suggesting these analyses were unlikely to be substantially biased by weak instruments (Table S4). Mean F statistics for EPA analyses were 9, leading to possible weak instrument bias.

### Major depressive disorder

**Fig. 4:**
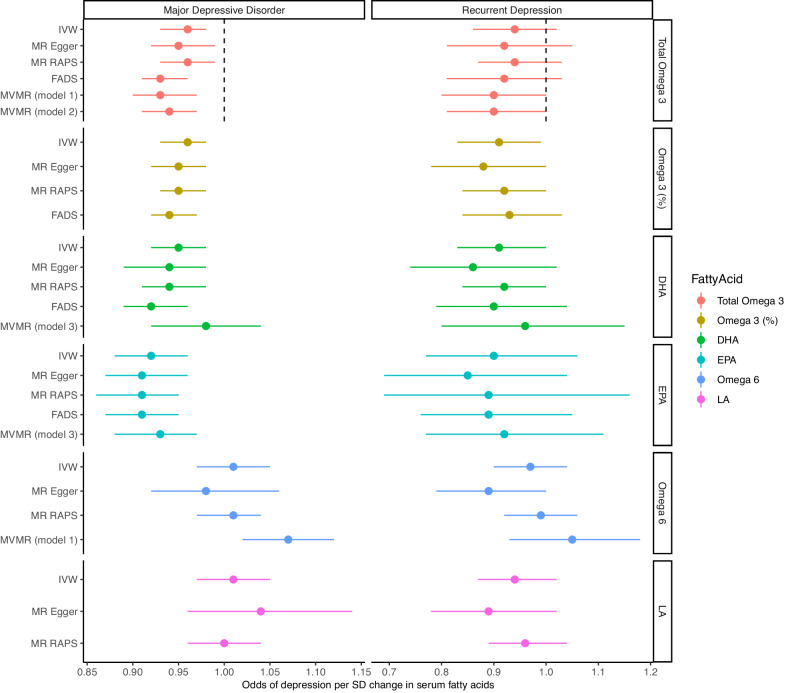
MR results for multiple fatty acid exposures on MDD (*n* = 480,539) and rMDD (*n* = 80,933). MR results for MDD and rMDD outcomes using different methods are shown for each fatty acid exposure. All exposure SNPs and SNP-exposure estimates are derived from UKBB, except EPA, which uses effect sizes derived from the CHARGE EPA GWAS [48] (see Supplementary Methods 1). MR estimates restricted to the *FADS* SNP have been omitted for LA and omega 6 due to the inconsistency between estimates derived from other MR methods, which suggest that the results may be driven by pleiotropy (see discussion for more details). In addition to being potentially misleading, the impact of the wide confidence intervals on-axis measurements hinders the visualization of remaining results.

Table 1 provides MR estimates for each fatty acid exposure and MDD outcome, with results depicted graphically in Fig. 4. Results were consistent with a protective effect of genetically elevated omega-3 on the risk of MDD. Point estimates for longer chain fatty acids EPA (OR_IVW_ 0.92 (95% CI: 0.88–0.96) *p* = 0.0002) and DHA (OR_IVW_ 0.95 (0.92–0.98) *p* = 0.001) were larger than for total omega-3 (OR_IVW_ 0.96 (0.93–0.98) *p* = 0.003), or omega-3 (%) (OR_IVW_ 0.96 (0.93–0.98) *p* = 0.0002), though confidence intervals overlapped.

**Table 1:** Results for two-sample Mendelian randomization using UKBB exposures and PGC MDD sample (without UKBB *n* = 480,359), and rMDD subsample (*n* = 80,933).

	nSNP	Method	Major Depressive Disorder (n=480,359) OR (95%CI)	*p*	nSNP	Recurrent Depression (n= 80,933) OR (95%CI)	*p*
*OMEGA-3*							
Total Omega-3	43	IVW	0.96 (0.93–0.98)	0.003	45	0.94 (0.86–1.02)	0.13
		Egger	0.95 (0.92–0.99)	0.02		0.92 (0.81–1.05)	0.24
		*MR-RAPS*	0.96 (0.93–0.99)	0.01		0.94 (0.87–1.03)	0.20
		*FADS* (rs174564)	0.93 (0.91–0.96)	1.3E−5		0.92 (0.81–1.03)	0.16
Omega-3%	33	IVW	0.96 (0.93–0.98)	0.0002	33	0.91 (0.83–0.99)	0.03
		Egger	0.95 (0.92–0.98)	0.002		0.88 (0.78–1.00)	0.07
		*MR-RAPS*	0.95 (0.93–0.98)	5.78E−5		0.92 (0.84–1.00)	0.05
		*FADS* (rs174564)	0.94 (0.92–0.97)	1.3E−5		0.93 (0.84–1.03)	0.16
DHA	40	IVW	0.95 (0.92–0.98)	0.001	40	0.91 (0.83–1.00)	0.06
		Egger	0.94 (0.89–0.98)	0.01		0.86 (0.74–1.02)	0.09
		*MR-RAPS*	0.94 (0.91–0.98)	0.001		0.92 (0.84–1.01)	0.09
		*FADS* (rs174564)	0.92 (0.89–0.96)	1.3E−5		0.91 (0.84–1.03)	0.16
EPA	39	IVW	0.92 (0.88– 0.96)	0.0002	38	0.91 (0.77– 1.08)	0.27
		Egger	0.91 (0.87–0.96)	0.002		0.85 (0.69–1.04)	0.13
		*MR-RAPS*	0.91 (0.86–0.95)	0.0001		0.89 (0.69–1.16)	0.39
		*FADS* (rs174564)	0.91 (0.87–0.95)	1.3E−5		0.89 (0.76–1.05)	0.16
*OMEGA-6*							
Omega-6	50	IVW	1.01 (0.97–1.05)	0.60	51	0.97 (0.90–1.04)	0.44
		Egger	1.01 (0.92–1.06)	0.68		0.89 (0.79 –1.00)	0.06
		*MR-RAPS*	1.01 (0.97–1.04)	0.66		0.99 (0.92–1.06)	0.70
		*FADS* (rs174564)	0.33 (0.21–0.5)	5.73E−7		0.17 (0.01–1.99)	0.16
LA	50	IVW	1.01 (0.97–1.05)	0.57	51	0.94 (0.87– 1.02)	0.12
		Egger	1.04 (0.96–1.14)	0.36		0.89 (0.78– 1.02)	0.09
		*MR-RAPS*	1.00 (0.96–1.04)	0.95		0.96 (0.89–1.04)	0.32
		*FADS* (rs174564)	1.32 (1.16– 1.49)	5.73E−7		1.42 (0.87– 2.29)	0.16

Odds ratios for MDD are given per standard deviation increase in exposure. EPA SNP-effect sizes are taken from CHARGE consortium GWAS and the smaller sample size is reflected in the *F* statistics. Further details on individual SNPs are given in supplementary Excel spreadsheet S3, measures of instrument strength and pleiotropy are provided in Supplementary Table S4. All SNPs in the analyses had a stronger effect on the exposure than the outcome, so repeat analyses with Steiger Filtering were identical. *FADS* gene analyses used a single SNP on chromosome 11 (*rs174564*). Further analyses, including weighted median and mode MR methods, and the comparison of results with and without the sample overlap for UKBB are contained in Supplementary Material S6.

Individual sensitivity plots for each fatty acid on MDD are presented in Supplementary Information (S5). Single SNP analyses suggested the *FADS* gene cluster was a major contributor to the observed effects of omega-3 on MDD. Omitting the *FADS* SNP (*rs174564*) in ‘leave one out’ analyses attenuated the effect sizes and widened the confidence intervals to include the null in all analyses, though the effect direction remained consistent, especially for the longer chain omega-3 fatty acids (OR_EPA_ 0.94 (0.86–1.10) *p* = 0.20, and OR_DHA_ 0.98 (0.93–1.03), *p* = 0.39, see S5). UKBB sample overlap did not alter point estimates, but as expected, confidence intervals were narrower using the complete PGC MDD sample (*n* = 807,553) compared to the sample with UKBB removed (*n* = 480,539, see S6).

We identified no evidence that genetically elevated omega-6 fatty acids altered MDD risk (OR_IVW_ per SD increase in total omega-6: 1.01 (95% CI 0.97–1.05), *p* = 0.60, and OR_IVW_ for LA 1.01 (0.97–1.05), *p* = 0.57) (see Table 1).

### Recurrent depression (rMDD)

MR analyses for rMDD were consistent with our primary analyses, although confidence intervals were wider, as expected from the smaller sample size. For each SD increase in total omega-3 the odds of rMDD decreased: OR_IVW_ 0.94 (95% CIs 0.86–1.02); *p* = 0.13), with similar findings for omega-3%: OR_IVW_ 0.91 (95% CIs 0.83–0.99); *p* = 0.03), DHA: OR_IVW_ 0.91 per SD (95% CIs 0.83–1.00); *p* = 0.06), and EPA: OR_IVW_ 0.91 per SD (95% CIs 0.77–1.08); *p* = 0.27). Although point estimates for omega-3 fatty acid measures were marginally larger for rMDD than for MDD, they were not statistically different.

### Consistency across methods and instruments

Results for all omega-3 analyses were consistent across MR methods, increasing confidence in the findings (see Table 1, Fig. 4, and individual scatter plots S5). Heterogeneity was substantial for most analyses (total omega-3 (*Q* = 55, *p* = 0.01), DHA (*Q* = 55, *p* = 0.05) omega-3% (*Q* = 33, *p* = 0.39) and EPA (*Q* = 63, *p* = 0.01). While we did not find evidence for directional pleiotropy—with Egger intercepts close to zero—a wide confidence interval meant we were unable to rule it out.

### Establishing directionality

As all omega-3 instruments used in the primary analyses explained greater variance in the exposure than the outcome, Steiger filtering did not remove any SNPs, and the results were unchanged. Reverse MR provided no evidence that genetic liability to MDD lowered circulating omega-3 levels (beta_IVW_ 0.03 SD per doubling of genetic risk (−0.02, 0.09), *p* = 0.23) (see Supplementary Table S7).

### Biological pathways

As the *FADS* SNP (*rs174564*) explained a large proportion of variance in the omega-3 measures, omega-3 analyses restricted to the single *FADS* SNP were similar in direction and magnitude to the multi-SNP analyses (see Table 1). Analyses restricted to the *FADS* SNP for total omega-6 suggested an inverse effect on both MDD (OR_*rs174564*_ = 0.33 (0.21–0.5), *p* = 5.73E^−7^) and rMDD (OR_*rs174564*_ = 0.17 (0.01–1.99, *p* = 0.16). The reverse was seen for LA, with a strong positive association between genetically increased LA and MDD (OR_*rs174564*_ = 1.32 (1.16–1.49), *p* = 5.73E^−7^), and rMDD (OR_*rs174564*_ = 1.42 (0.87–2.29, *p* = 0.16 (Table 1)).

### Multivariable models

Effect estimates for total omega-3 fatty acids were not altered by MVMR analyses when adjusting for other lipids (Table 2 and Fig. 4). In model 1, which accounted for total omega-6, the OR_IVW_ estimate for total omega-3 was 0.93 (0.90–0.97), *p* = 0.0001. In the second model accounting for triglycerides and cholesterol, the was unchanged from the univariable analyses (OR_IVW_ 0.96 (0.93–0.98), *p* = 0.001).

**Table 2:** Multivariable MR analyses.

	Exposure	GWAS	nSNP	OR	*p*	Conditional *F*	*Q* (*p*)
Model 1	Omega-3 fatty acids	met-d-Omega_3 [49]	43	0.93 (0.90–0.97)	0.0001	268	96 (0.01)
	Omega-6 fatty acids	met-d-Omega_6 [49]	49	1.07 (1.02–1.12)	0.01	165	
Model 2	Triglycerides	ieu-b-111 [50]	388	1.08 (1.03–1.13)	0.002	30	682 (9.88E−18)
	HDL cholesterol	Ieu-b-109 [50]	387	1.06 (1.01–1.10)	0.01	42	
	LDL cholesterol	Ieu-b-110 [50]	388	0.98 (0.93–1.03)	0.37	43	
	Omega-3 fatty acids	met-d-Omega_3 [49]	387	0.96 (0.93–0.98)	0.001	25	
Model 3	EPA	Lemaitre [48]	40	0.93(0.88–0.97)	0.002	9	52 (0.05)
	DHA			0.98 (0.92–1.04)	0.46	6	

In MVMR model 3, point estimates for EPA were larger than for DHA, and while adjusting for DHA made little change to the effect size for EPA (MVMR OR_EPA_0.93 (0.88–0.97), *p* = 0.002), estimates for DHA were attenuated when adjusting for EPA (MVMR OR_DHA_ 0.98 (0.92–1.04), *p* = 0.46, see Table 1, and Fig. 4).

As *Q*-statistics for each MVMR model were suggestive of high heterogeneity (*p* ≤ 0.05), and conditional *F* statistics for DHA and EPA were <10, further MVMR analyses were undertaken to confirm findings (see Supplementary Material S8, Table S8a, b, and c). The additional analyses used alternative GWAS studies for either the identification of instruments, SNP-exposure estimates, or both. Further analyses supported the findings in our main analyses, with improvements in heterogeneity discussed.

### Colocalization

Colocalization using our primary outcome sample was supportive of a shared causal variant between omega-3 measures and MDD (PPA = 88.9% for omega-3 and DHA, and 97.1% for EPA; see Supplementary Table S9a and Fig. S9b). However, this was not the case when using the sample including UKBB, where the probability for distinct causal variants increased (PPA_H4_ 27.4% vs. PPA_H3_ 72.5% for Omega-3 and PPA_H4_ 16.3% vs. PPA_H3_83.6% for EPA.) The strongest regional signal for MDD in the sample, including UKBB, was located on a neighbouring gene (*DAGLA*, r*s198457*), which is in partial LD with the *FADS* SNP (*rs174564: rs198457* *r*2 = 0.10). These results could be explained either by confounding by LD or a violation of colocalization’s assumptions due to the presence of multiple causal variants in the region. Excessive prior variance in the outcome prevented the use of methods relaxing the single variant assumption (see Supplementary Material S9).

### PheWAS

Over 400 traits were identified in the PheWAS of the lead SNP in the analyses (*rs174564)*, although many of these studies were either highly correlated or repeated measures (see Supplementary Material S10). As expected for an SNP encoding an important enzyme for lipid metabolism, most phenotypes were lipid measures, with other metabolic, endocrine and haematological measures the next most prevalent. The strongest of the lipid measures were included in MVMR models to account for potential bias due to horizontal pleiotropy.

## Discussion

Our results suggest a protective effect of multiple genetically predicted omega-3 fatty acid measures on MDD and its recurrence, with no evidence that this is driven by the effect of MDD on circulating omega-3. There was no evidence that genetically elevated omega-6 fatty acids altered MDD risk overall, except in MR analyses restricted to the *FADS* SNP, which may be biased by pleiotropy, as discussed below. Effect sizes are modest, particularly as they reflect a lifetime of exposure. Our results are consistent with point estimates from previous MR studies of omega-3 fatty acids in MDD, which showed similar magnitudes of effect and consistency across methods but lacked statistical power [22, 23]. Large GWAS samples of both fatty acids and MDD facilitated the detection of modest causal effects, emphasising the power of increasing genetic sample sizes for nutritional psychiatry MR.

As with all methods, MR has its limitations and assumptions, which we have attempted to mitigate. One limitation is the sample overlap between our exposure measures and our rMDD outcome, as both were UKBB GWASs. Recent studies have downplayed the importance of sample overlap, especially with large outcome samples [37, 38]. As a sensitivity analysis, we checked whether the MR estimate for our primary outcome (MDD) was affected by using a GWAS with UKBB data included. As MR results were unaffected, bias is likely to be minimal (see S6). Furthermore, effect sizes for EPA were extracted from the CHARGE consortium, with consistent results.

The *FADS* gene cluster appeared to be a strong driver of the effect. Previous research has explored the role of the *FADS* gene cluster in schizophrenia [33], response to omega-3 supplementation [39] and infant cognition [40]. Studies investigating the *FADS* genotype in MDD have been inconclusive, though sample sizes have been comparatively small [41, 42]. On the one hand, this adds weight to the biological plausibility of causal inference, being involved in a rate-limiting step in the elongation of short-chain fatty acids to the long-chain derivatives important for brain health (such as EPA and DHA). However, as the *FADS* gene cluster affects multiple fatty acids and other complex lipid metabolic processes, [6] it is difficult to rule out horizontal pleiotropy, in which the observed MR effect is due to alternative pathways. An example of pleiotropy is demonstrated by the total omega-6 and LA analyses restricted to the *FADS* variant (*rs174564)*, which suggest a risk-increasing effect for LA and a protective effect for total omega-6. However, in the context of multi-SNP and MVMR analyses, it is likely that these results reflect diminished activity of desaturase enzymes rather than directly adverse effects of LA. Genetic variation that reduces desaturase enzyme activity will have numerous metabolic consequences, including reduced long-chain fatty acid synthesis, as well as increased short-chain fatty acid precursors such as LA (see Fig. 1). Taken in isolation, the *FADS* SNP analyses are unable to identify which fatty acid pathways are driving the effect on MDD, and which are due to alternative mechanisms, highlighting the importance of interpreting MR results in context. To investigate whether pleiotropy may underlie the association between long-chain omega-3 fatty acids and MDD, we used MVMR models to account for other lipids, which preserved a strong effect of omega-3 on MDD. Our supplementary PheWAS of the lead *FADS* SNP identified many potential sources of pleiotropy. However, some of these may be downstream effects of insufficient omega-3, in which case conclusions remain valid. Studies showing improved lipid profiles among omega-3-supplemented animals and the introduction of EPA treatments to reduce hypertriglyceridemia in humans may support this [43]. Colocalization analyses were suggestive of a shared causal variant between omega-3 and MDD for our primary outcome sample, though not for all outcomes. This may be due to multiple conditionally independent SNPs in the region violating the assumptions of colocalization, or it could be confounding by linkage disequilibrium driving the MR results.

Our results tentatively support the role of omega-3 fatty acids in the aetiology of MDD and justify further research into underlying mechanisms. Our MVMR estimate, suggesting a stronger effect for EPA than DHA, is consistent with meta-analytical evidence for EPA-predominant formulations [16] and may explain differential trial outcomes between omega-3 formulations with insufficient EPA doses or EPA:DHA ratios. Pure DHA supplements in animal studies appear to slow the hepatic conversion of EPA into its metabolic products [44], with theoretically adverse effects on MDD risk if a protective effect is EPA-driven. Assuming a differential effect of EPA and DHA on MDD may also facilitate a more targeted exploration of underlying mechanisms, with systemic inflammation a possible candidate. Many EPA derivatives are anti-inflammatory, as well as competitively inhibiting pro-inflammatory omega-6 derivatives (see Fig. 1). Studies have identified differential anti-inflammatory effects from EPA and DHA supplementation, exploration of which may extend current mechanistic understanding [45, 46]. A central effect of omega-fatty acids in the brain is also possible, whether directly through effects on cellular membrane properties, neurotransmitter release, or via neuroinflammatory processes [45]. While DHA is the predominant omega-3 in the brain, eicosanoid derivatives of EPA are found in brain tissue and may moderate neuroinflammation. In contrast to other brain cells, EPA concentrations within microglial cells, the resident macrophages within the brain, appear to outweigh DHA. There is evidence that omega-3 fatty acids reduce microglial activation, affecting cytokine production that may impact neurogenesis, neuroplasticity, as well as neurotransmitter metabolism [47].

Further MR analyses could consider the differential effects of EPA and DHA on potential mediators, such as inflammation, which may consolidate this hypothesis and strengthen the rationale for high-dose EPA interventions. Given relatively small effect sizes, future trials could consider targeting participants with sub-optimal long-chain omega-3 fatty acid intake, high omega-6:3 ratios, or genetically low- conversion of short- to long-chain fatty acids, which might yield greater benefits and require smaller sample sizes than universal prevention efforts. Further one-sample MR studies could also investigate omega-3 fatty acid toxicity at higher concentrations by using non-linear methods, as well as whether this modest effect masks a larger threshold effect by the inherent assumption of a linear relationship in two-sample MR. Finally, in the era of environmental awareness, the question of how to generate affordable and sustainable long-chain omega-3 fatty acids for a growing global population seems pertinent.

## Conclusion

Our results provide evidence for a link between genetically predicted omega-3 fatty acids and MDD. The effect appears strongest for EPA, remains robust to biologically correlated lipids, and is not explained by reverse causality. This strengthens the evidence for a causal effect of omega-3 fatty acids on MDD, although horizontal pleiotropy and confounding by linkage disequilibrium cannot be fully excluded. Further research to triangulate these findings and consider potential mediating mechanisms and phenotypes would be valuable. Future trials of omega-3 supplementation for MDD should ensure adequate power to detect modest effects, along with ensuring formulation, dose, and participant selection are the most likely to benefit.

### Supplementary information

Supplementary Material Document

Supplementary Material S2

Supplementary Material S3

Supplementary Material S10

## Data Availability

The genetic instruments for Omega fatty acids used in these analyses are contained in the supplementary material. Full GWAS summary statistics for UK Biobank fatty acids exposures are available online from the IEU Open GWAS project (https://gwas.mrcieu.ac.uk) [29]. Summary statistics for the CHARGE consortium (GWAS studies of EPA and DHA) can be downloaded directly from the CHARGE consortium website (https://web.chargeconsortium.com/main/results). Further information about obtaining access to the PGC summary statistics can be found at: http://www.med.unc.edu/pgc/statgen.
