# Supplementary material for: Omega-3 fatty acids and major depression: a Mendelian randomization study
Source: Transl Psychiatry. 2024 May 29;14:222. doi: 10.1038/s41398-024-02932-w (PMC11136966; doi:10.1038/s41398-024-02932-w)
Supplement: Supplementary file 1 — Supplementary Material Document [file 41398_2024_2932_MOESM1_ESM.docx]

Omega & MDD MR: Supplementary Materials

Table of Contents

[S1. Supplementary Methods 1](#_Toc166147955)

[a. Assessing instrument strength and statistical power 1](#_Toc166147956)

[b. Additional Methods specific to CHARGE Exposure Data 2](#_Toc166147957)

[c. Validation Methods 2](#_Toc166147958)

[S2. Data Sources 6](#_Toc166147959)

[S3. SNP- exposure associations for Omega fatty acids from UKBB 6](#_Toc166147960)

[S4. Heterogeneity statistics for MDD analyses. 7](#_Toc166147961)

[S5. Individual MR sensitivity plots for causal effects of specific fatty acid exposures on MDD 8](#_Toc166147962)

[a. Total Omega 3 8](#_Toc166147963)

[b. Omega 3(%) 9](#_Toc166147964)

[c. DHA 10](#_Toc166147965)

[d. Omega 6 11](#_Toc166147966)

[e. LA 12](#_Toc166147967)

[f. EPA 13](#_Toc166147968)

[S6. Comparison between MR analyses using complete MDD outcome sample (n=807,553), and MDD sample removing UKBB sample overlap (n=480,359) 14](#_Toc166147969)

[S7. Reverse MR results 15](#_Toc166147970)

[S8. Comparison of multivariable MR models using different instruments. 16](#_Toc166147971)

[a. Model 1 16](#_Toc166147972)

[b. Model 2 17](#_Toc166147973)

[c. Model 3 18](#_Toc166147974)

[Fig S8d 19](#_Toc166147975)

[S9. Colocalization 21](#_Toc166147976)

[S10. Phewas results 23](#_Toc166147977)

[Supplement References 24](#_Toc166147978)

## S1. Supplementary Methods

### Assessing instrument strength and statistical power

#### Calculating variance

The variance explained in our exposure by each SNP was calculated by using the GWAS effect size (*beta),* effect allele frequency (EAF), standard error (SE), and sample size (N) for each SNP using the following equation:^1^

2 x beta^2^x EAF x (1-EAF) / (2 x beta^2^ x EAF x (1-EAF) + SE^2^ x 2 x N x EAF x (1-EAF))

The total variance explained in each exposure (R^2^) was calculated as the sum of individual SNP variances.

#### Instrument Strength (F)

F-statistics for each exposure were calculated using the formula:

F= (Σ r^2^/nSNP)/ ((1-Σ r^2^)/(N-nSNP-1))

Where Σ r^2^ is the sum of the variance explained in the exposure for each SNP, nSNP is the number of SNPs and N is the sample size of the exposure GWAS instrument strength was quantified using the mean F statistic ($\overline{F}$)^2^, and the conditional *F* statistic^3, 4^ for MVMR analyses. We considered the conventional threshold ($\overline{F}$ <10) as weak instruments for our exposure of interest, and at risk of bias.

#### Statistical Power

For each fatty acid exposure, we estimated the minimum effect size (OR_min_) for which we had over >80% power to detect based on available data, using an online power calculator for MR studies (https://shiny.cnsgenomics.com/mRnd/).^5^ We used the estimated proportion of variance (R^2^) in each fatty acid explained by our genetic instruments (see S2), along with our MDD sample size (n=408,359), proportion of cases (0.3), and type-I error rate (α=0.05). All MDD analyses had power to detect OR_min_ ≤ 0.95 (or ≥ 1.05) per SD increase. Given the smaller rMDD sample size (n=80,933) we were able to detect an OR_min_ ≤ 0.90 (or ≥ 1.10) per SD increase in UKBB measures, and OR_min_ ≤ 0.89 (or ≥ 1.11) per SD increase in EPA.

### Additional Methods specific to CHARGE Exposure Data

#### Identifying Instruments

As the GWAS study of EPA in the Cohorts for Heart and Aging Research in Genomic Epidemiology (CHARGE) Consortium were on a much smaller sample (n=8,866), only 2 SNPs for EPA remained after clumping, prohibiting the use of sensitivity analyses requiring more SNPs. We therefore took the instruments identified from the UKBB total omega 3 GWAS and extracted the SNP-EPA effect sizes from the EPA GWAS study. For the multivariable analysis of EPA and DHA, conditional F statistics were low using UKBB SNPs (F_EPA_ =9, and F_DHA_ =6). We confirmed the findings using instruments derived from a previous GWAS of total omega 3 by Kettunen et al.^6^ 5 genome wide significant SNPs were identified from this GWAS, and the SNP-exposure effect sizes were extracted from the CHARGE consortium EPA and DHA GWAS summary statistics. Conditional F statistics were stronger for this analysis, and hence are reported in the main paper, with the details of supplementary MVMR analysis in the supplementary S8 (‘MVMR.xls’).

#### Standardizing Effect Estimates

As CHARGE Consortium GWAS effect estimates are presented as % of total fatty acids- they were converted to standardized units (SD (mmol/L)) to help interpretation. This was achieved using GWIS (Genome-wide Inferred Study),^7^ a method that approximates GWAS summary statistics from phenotypes for which GWAS summary statistics, phenotypic means, and covariances are available. SD (mmol/L) SNP-effect estimates were derived as a linear function of the allele frequencies, population means of the fatty acid concentrations, and SNP-% fatty acid effect estimates.

### Validation Methods

Mendelian Randomization can estimate the effect of an exposure on an outcome providing certain assumptions are met.^8^ These assumptions are:

1. That is that the genetic variant (i.e. SNP) is strongly associated with the exposure of interest,
2. That an association between the SNP and the outcome results from changing the exposure, and
3. That the SNP isn’t associated with the outcome through potentially confounding pathways.

To increase the confidence in the validity of our findings, we used several sensitivity analyses to check for any invalidation of these assumptions. For the first assumption we used stringent GWAS p value thresholds (<5e^-8^) to identify genetic instruments, and estimated instrument strength (F) as described below. For the second assumption, we used a selection of MR methods, as each make different assumptions: the IVW method has the greatest statistical power, but can be biased in the presence of pleiotopy; MR Egger is less efficient but is able to identify unbalanced horizontal pleiotropy (i.e. where the SNP affects the outcome through an alternative pathway); and MR RAPS which reduces the bias from outlying SNPs and improves estimates in the presence of many weak instruments. Consistency across these different methods increases the strength of causal evidence. To further investigate the second two assumptions, we undertook further sensitivity analyses as described below.

#### Effect Direction

We used two methods to confirm that the association between the SNPs and MDD resulted from an effect on the exposure, and not the reverse. Firstly, we used ‘Steiger filtering’^9^ to retain only SNPs explaining a greater amount of variance in the exposure than the outcome, as would be expected for a true causal effect in this direction. Secondly, we used ‘reverse MR’ to consider whether genetic liability to MDD affected circulating omega 3, potentially causing a spurious result. For MR in the reverse direction, we identified genome wide significant SNPs (p<5x10^-8^) from the PGC MDD GWAS (minus UKBB samples). After clumping (r2<0.001) 27 MDD SNPs remained. Outcome data for these 27 SNPs were then extracted from the total omega-3 and DHA UKBB GWASs, and MR undertaken using the TwoSampleMR package as described above.

#### Biological Plausibility and Potential Pleiotropy

To investigate the biological plausibility of the effect, we restricted MR to biologically defined pathways relating to omega-3 fatty acid biosynthesis, using only the strongest single SNP within the *FADS* gene cluster on chromosome 11 (*rs174564*), and the *ELOVL2* gene on chromosome 6 (*rs2295602*). These genes code enzymes that convert shorter chain fatty acids (derived from dietary sources, such as alpha-linoleic acid (ALA)), into longer chain fatty acids (such as EPA and DHA,) thought to be the most relevant biological omega-3 fatty acids for the development of MDD.

Multivariable MR is a method used to account for genetically correlated traits in MR, and establish the direct effect of an exposure, after accounting for the effect of the instruments on genetically correlated exposures.^10^ We used the TwoSampleMR package mv_multiple function to estimate the direct effects of omega-3 FA after accounting for correlated traits. MVMR models were limited to a few simultaneous exposures, as an increasing number of exposures reduced the conditional F statistics affecting the precision of our estimates, and also because weak instruments in MVMR can lead to bias in unpredictable directions.^11^ To calculate conditional F statistics, we used the PhenoSpD R package(ref) to estimate the correlation between phenotypes using summary statistics,, and input the covariance matrices into the MVMR package.^11^

We fitted three MVMR models (see table 2). The first model looked at differential effects between omega 3 and omega 6 fatty acids, using SNPs identified from our primary analyses.

In the second model, we included the commonly measured lipids in clinical practice- triglycerides, high-density lipoprotein (HDL) and low-density lipoprotein (LDL) cholesterol- to establish a direct effect of omega-3 fatty acids on MDD, adjusting for broader lipid profile. Genome wide significant SNPs for triglycerides, HDL and LDL cholesterol were obtained from a GWAS study of these exposures among UKBB participants.^12^

In the third model we considered whether there was a difference in effect between EPA and DHA on MDD, using effect sizes for each exposure derived from the CHARGE consortium.^13^ As MVMR models using SNP-exposure effect sizes of UKBB instruments gave poor conditional F statistics and high heterogeneity (model 3a), we compared these results with MVMR models using alternative instruments. In model 3b, SNPs were selected from a GWAS study of total omega 3, as EPA was unavailable. In both models the SNP-exposure effects were extracted from the relevant CHARGE GWAS. We used a pairwise t-test to calculate the likelihood that the difference between the observed effect sizes for each exposure in the model had occurred by chance.

#### Exploring Biological Pathways - FADS and ELOVL Analyses

To explore biological mechanisms, we identified SNPs for the *FADS* gene cluster and *ELOVL2* gene which encode key desaturase and elongase enzymes involved in omega-3 and omega-6 fatty acid biosynthesis pathways. SNP-exposure data for SNPs within the *FADS* gene cluster locus (chr11:61560452-61659523 ± 500kb) and *ELOVL2* gene locus (chr6:10980992-11044624 ± 500kb) were extracted from the UKBB DHA GWAS. SNP-outcome data was then extracted from the MDD GWAS, followed by LD clumping to identify the single strongest SNP for each genetic locus. As no SNPs on the *ELOVL2* gene were strongly associated with UKBB GWAS Omega-3 or -6 measures, none of the SNPs identified through this approach gave satisfactory F statistics, leading to extreme confidence intervals and highly variable point estimates. We therefore have omitted *ELOVL2* analyses.

#### Colocalization

**Linkage disequilibrium (LD) could lead to biased results in Mendelian randomization if, by chance, the selected genetic instrument influencing fatty acids is** correlated (i.e. in LD) with another genetic variant influencing the risk of MDD independently. We used the ‘*coloc’* R package,^14^ a method for pairwise genetic colocalization analysis, to estimate the posterior probability for the association with both traits being explained by the same causal variant in the FADS region. To do this, we selected the top SNP in the *FADS* region (*rs174564),* and all SNPs within a 500kbp radius. We used the ‘*gassocplot’* R package to plot the SNPs for total omega 3, EPA and DHA, along with MDD, rMDD and the MDD sample including UKBB.

Colocalization methods apply Bayesian probabilities to the SNP-trait associations of all SNPs within a defined region, to establish the likelihood that two traits are genetically linked. The *coloc* package in R uses prior probabilities that a SNP is associated with a trait (with a default of 1E^-4^) for each trait, and presents four distinct posterior probabilities of association (PPA):

H0 There is no causal variant for either trait in the region

H1 Causal variant for trait 1 only

H2 Causal variant for trait 2 only

H3 Both traits are associated, but have distinct causal variants

H4 Both traits are associated and share a single common causal variant

The *coloc* package provides a ‘posterior probability of association’ (PPA), with ≥ 70% (PPA for H4) considered as suggestive of a shared single causal variant between omega-3 fatty acids and MDD risk. This method assumes that a single common genetic variant exists for both traits, which may be unrealistic. An extension to this method ‘runsusie’ relaxes the single causal variant assumption,^15^ however, excessive prior probability in the outcome, (possibly as a result of the binary outcome measure, inherent to large scale psychiatric genomics research,) prohibited its use.

Results for the colocalization analyses are presented in S9: Colocalization, below.

#### PheWAS

As a supplementary, post-hoc analysis, we used the ieugwasr R package to undertake a Phenome Wide Association Study ‘PheWAS’ of the *FADS* SNP driving the apparent causal effect on depression (*rs174564).* A PheWAS is a technique used to scan multiple GWAS studies for association of a given SNP with multiple different phenotypes, to identify other potential phenotypes of interest. For our analyses, the PheWAS served two purposes. The first was to confirm variables included within our MVMR models were relevant, and the second was to consider potential mediating mechanisms and intermediate phenotypes on the biological pathway between omega-3 fatty acids and MDD, to facilitate further mechanistic research.

The *ieugwasr* package in R scans all traits contained within the IEU Open GWAS Database,^16^ which contains 14,582 harmonized GWAS datasets across a range of phenotypes and disease outcomes. By nature of the data, there are often multiple GWAS studies for traits (for example blood pressure, pulse rate or obesity). We used the default p-value threshold given in the ieugwasr package (p<5e-5). There are multiple highly correlated phenotypes that appear as individual ‘hits’. As the *FADS2* SNP contained many correlated traits relating to lipid metabolism, we have presented this as a separate category.

Results for the PheWAS are attached as the supplementary material S11, with an annotated plot of some key associations shown in S10 below.

## S2. Data Sources

Tables of data sources for exposure and outcome GWAS’s, including accessibility links and caveats are included in an excel spreadsheet “S2 Omega 3 supplement tables GWAS datasets.xlsx”.

## S3. SNP- exposure associations for Omega fatty acids from UKBB

Full SNP-exposure associations for individual exposures are included in “S3 SNP sets.xlsx” including details of the SNPs used for each exposure, effect alleles, estimated variance explained and Steiger p values for the MR analysis with MDD. SNP-exposure associations for total omega 3 fatty acids, total omega 6 fatty acids, DHA, LA and omega 3% were selected from the UKBB GWAS^17^ using the ieugwasr:: R package. Instruments for analyses of EPA (and DHA in MVMR model 3), were identified from the UKBB total Omega 3 GWAS, and SNP-exposure associations were extracted from the relevant CHARGE consortium GWAS study.^13^

## S4. Heterogeneity statistics for MDD analyses.

Heterogeneity statistics and mean instrument strength ($\overline{F})$ for each exposure are given below. The *FADS* SNP (rs174564) explained a large proportion of the variance in all exposures, with mean F statistics between 15- 6,315. Due to the smaller EPA GWAS sample size, mean F statistics for EPA analyses were 9, introducing possible weak instrument bias.

| **Exposure** |  | **GWAS**  **mean (SD)** | **nSNPs** | **Variance explained (%)** | $\overline{F}$ | **Cochran’s Q (p)** | **Rucker’s Q (p)** | **Egger Intercept (p)** |  |
| --- | --- | --- | --- | --- | --- | --- | --- | --- | --- |
|  |  |  |  |  |  |  |  |  |  |
| **Total Omega 3** | |  | 0.53 mmol/l (0.22) | 43 | 9.84 | 241 | 65 (0.01) | 64 (0.01) | 0.001 (0.71) |
|  |  |  | *FADS* | 5.20 | 6315 | NA | NA | NA |  |
|  |  |  |  |  |  |  |  |  |  |
| **Omega 3 %** |  | 4.42% (1.56) | 33 | 9.81 | 316 | 33 (0.39) | 33 (0.37) | 0.001 (0.47) |  |
|  |  |  | FADS | 6.93 | 8572 | NA | NA | NA |  |
|  |  |  |  |  |  |  |  |  |  |
| **DHA** |  | 0.23 mmol/l (0.08) | 40 | 7.47 | 189 | 55 (0.05) | 54 (0.04) | 0.002 (0.53) |  |
|  |  |  | FADS | 4.20 | 4826 | NA | NA | NA |  |
|  |  |  |  |  |  |  |  |  |  |
| **EPA** |  | 0.67% (0.41) | 39 | 3.77 | 9 | 63 (0.01) | 60 (0.01) | 0.001 (0.16) |  |
|  |  |  | *FADS* | 2.91 | 266 | NA | NA | NA |  |
|  |  |  |  |  |  |  |  |  |  |
|  |  |  |  |  |  |  |  |  |  |
| **Total Omega 6** |  | 4.45 mmol/l (0.68) | 50 | 6.31 | 120 | 62 (0.11) | 61 (0.10) | 0.001 (0.44) |  |
|  |  |  | FADS | 0.01 | 15 | NA | NA | NA |  |
|  |  |  |  |  |  |  |  |  |  |
| **LA** |  | 3.41mmol/l (0.69) | 42 | 4.75 | 124 | 61 (0.02) | 60 (0.02) | -0.002 (0.46) |  |
|  |  |  | FADS | 0.34 | 394 | NA | NA | NA |  |
|  |  |  |  |  |  |  |  |  |  |
| **Reverse MR** |  |  |  |  |  |  |  |  |  |
|  |  |  |  |  |  |  |  |  |  |
| **MDD** |  | NA | 25 | 0.87 | 37 | 25 (0.39) | 23 (0.48) | 0.005 (0.12) |  |
|  |  |  |  |  |  |  |  |  |  |

## S5. Individual MR sensitivity plots for causal effects of specific fatty acid exposures on MDD

For each exposure, the following plots are shown:

i) Scatter plot showing how MR estimates compare between MR methods.

ii) Funnel plot depicting instrumental variable precision. The log(odds ratios) of each IV is plotted on the x-axis (β_IV_) against instrument strength on the y axis (1/ SE_IV_). Asymmetry may suggest directional pleiotropy.

iii) Forest plot showing individual SNP ratio estimates (SNP-outcome estimate / SNP-exposure estimate), and

iv) Leave one out plot showing inverse variance weighted (IVW) estimates after omitting each SNP

### Total Omega 3


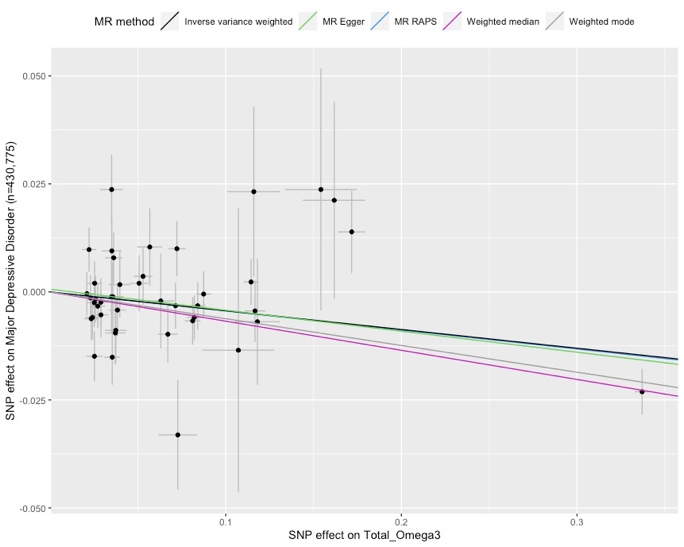

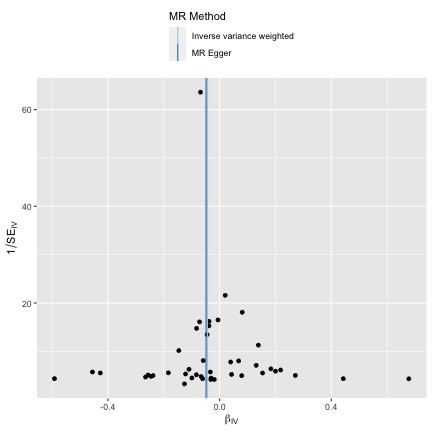

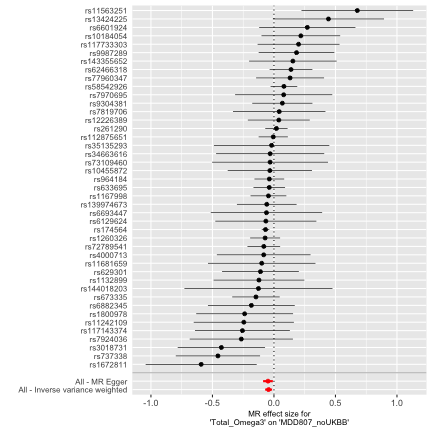

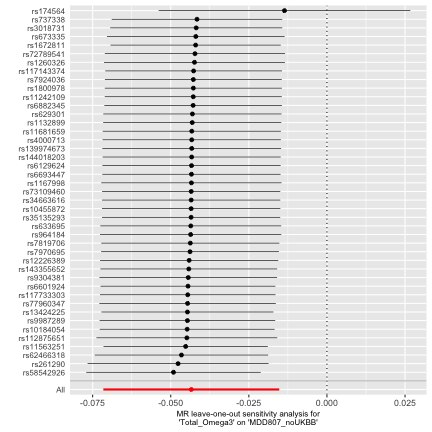


### Omega 3(%)


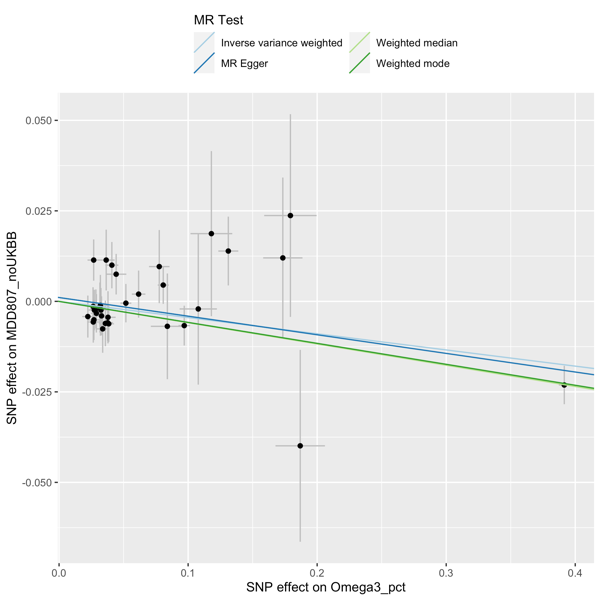

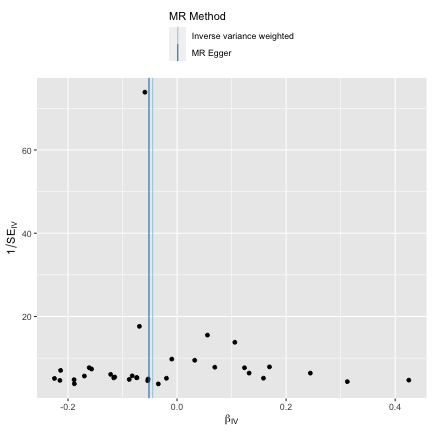

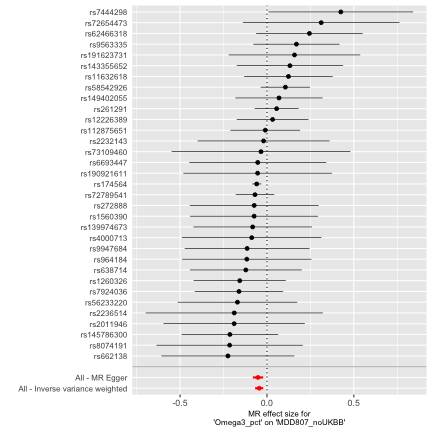

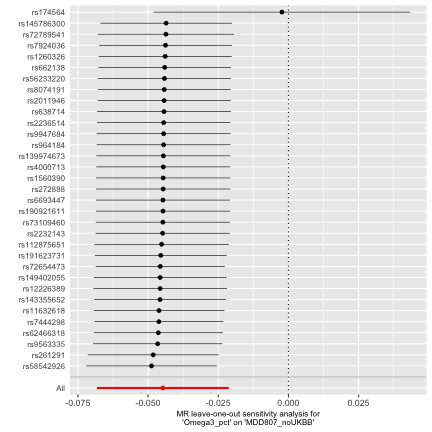


### DHA


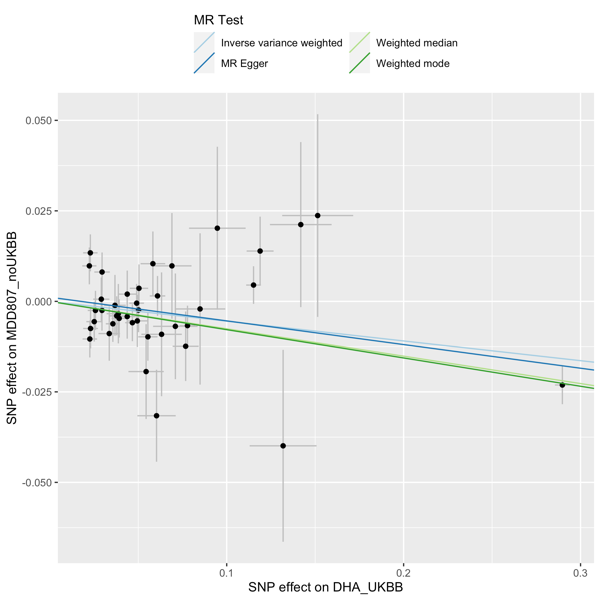

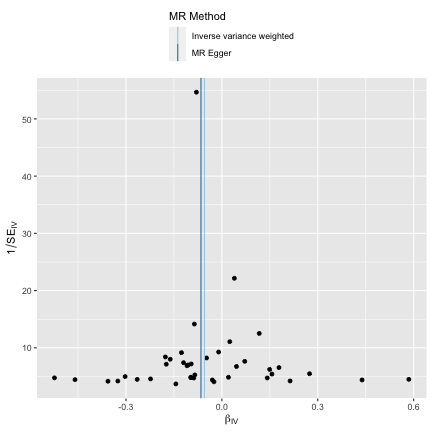

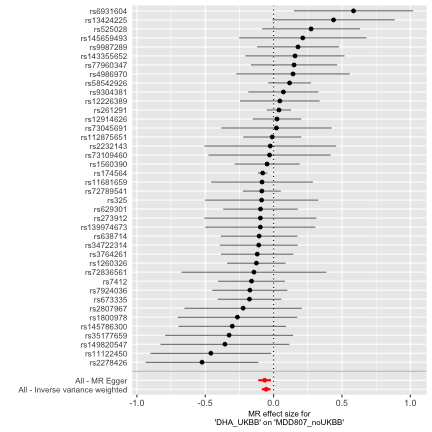

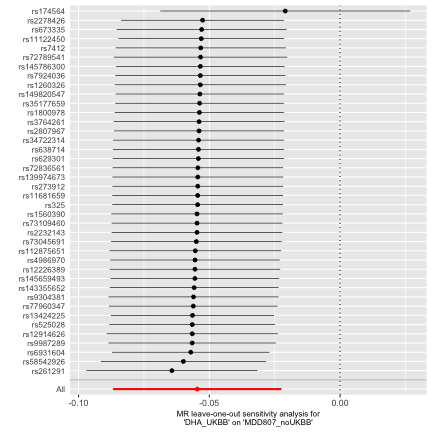


### Omega 6


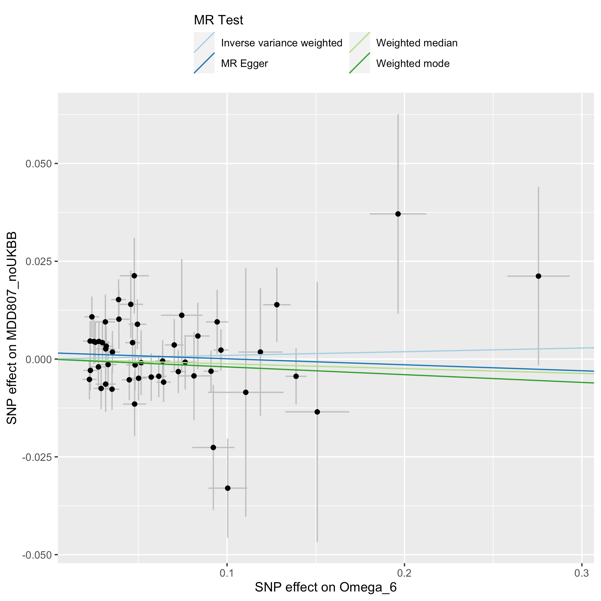

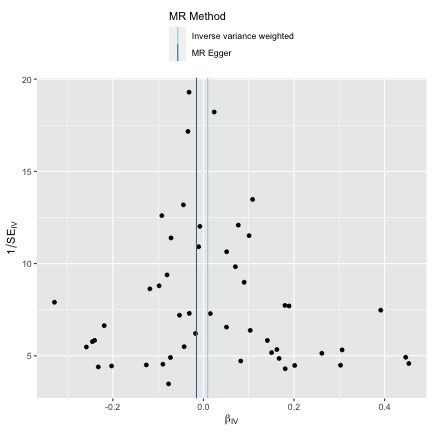

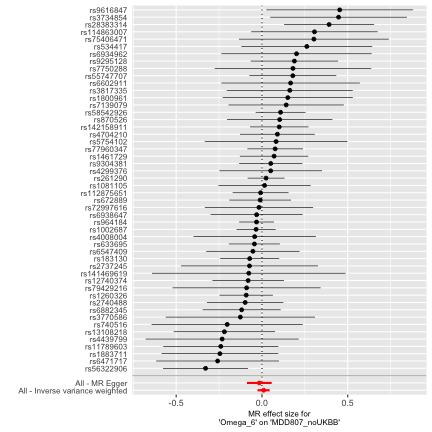

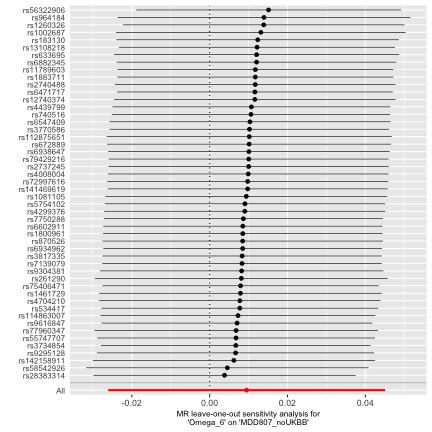


### LA


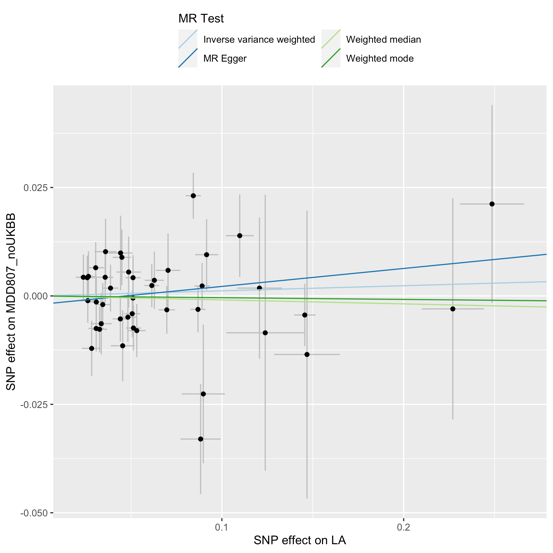

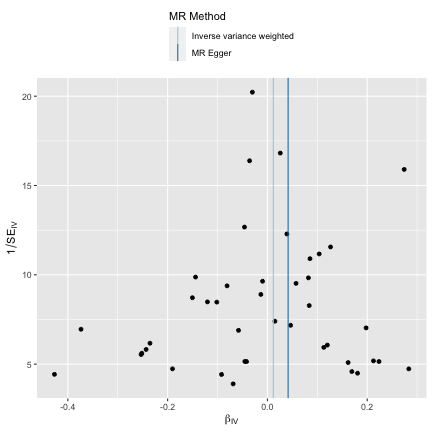


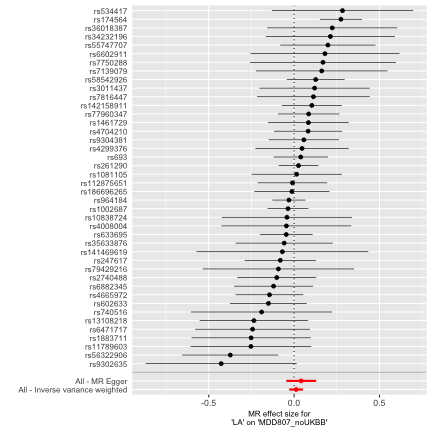

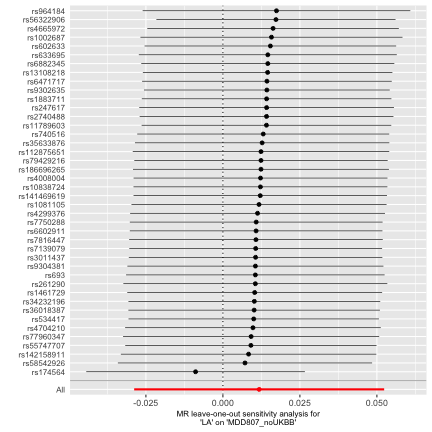


### EPA

As EPA is not measured in UK Biobank, these analyses use instruments identified from UK Biobank GWAS of Total Omega 3, with SNP-effect sizes taken from the Cohorts for Heart and Aging Consortium (CHARGE)^18^


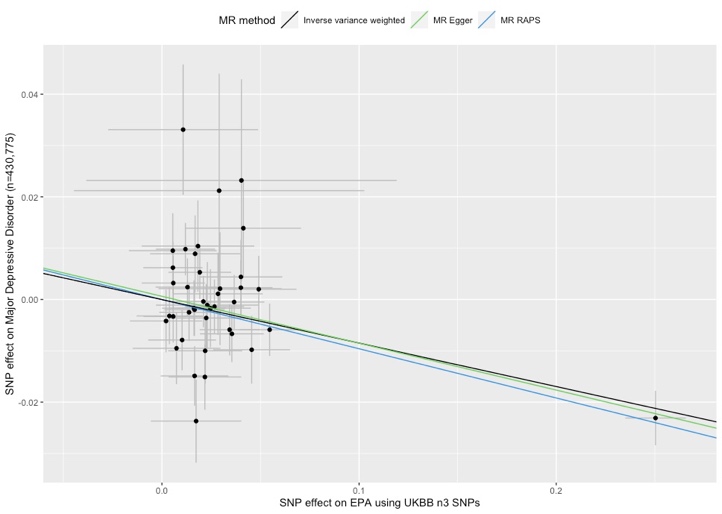

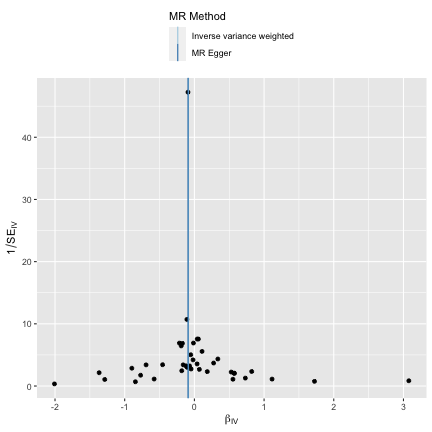


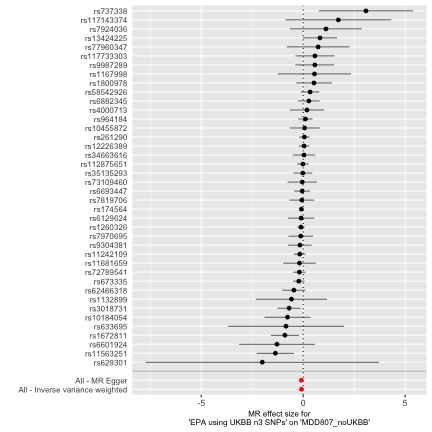

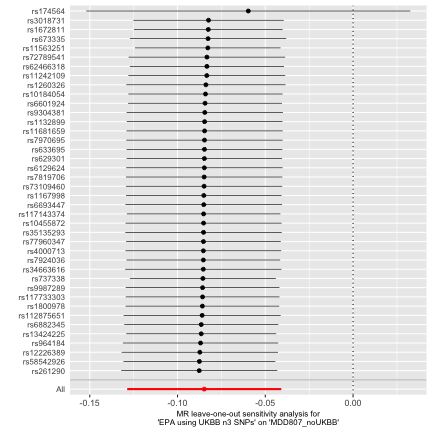


S6. Comparison between MR analyses using complete MDD outcome sample (n=807,553), and MDD sample removing UKBB sample overlap (n=480,359)

As a large proportion of the variation in omega-3 fatty acids was explained by the *FADS* SNP, weighted median and mode results were largely reflective of the *FADS* Wald ratio and are not presented in the main paper for simplicity.

|  |  |  |  | **MDD with UKBB removed (n=480,359)** |  |  | **MDD inc. UKBB (n=807,553)** |  |  |
| --- | --- | --- | --- | --- | --- | --- | --- | --- | --- |
|  | **nSNP** | **Method** |  | **OR (95%CI)** | **p** |  | **OR (95%CI)** | **p** |  |
|  |  |  |  |  |  |  |  |  |  |
| **OMEGA 3** |  |  |  |  |  |  |  |  |  |
|  |  |  |  |  |  |  |  |  |  |
| **Total Omega 3** | 43 | IVW |  | 0.96 (0.93-0.98) | 0.003 | 42 | 0.96 (0.95 -0.98) | 0.0005 |  |
|  |  | Egger |  | 0.95(0.92-0.99) | 0.02 |  | 0.96 (0.93 -0.99) | 0.007 |  |
|  |  | Weighted Median |  | 0.93 (0.92- 0.96) | 0.00001 |  | 0.95 (0.93 -0.97) | 0.0000009 |  |
|  |  | Weighted Mode |  | 0.94 (0.91 -0.97) | 0.0001 |  | 0.95 (0.93 -0.97) | 0.00004 |  |
|  |  |  |  |  |  |  |  |  |  |
|  |  |  |  |  |  |  |  |  |  |
| **Omega 3 %** | 33 | IVW |  | 0.96 (0.93-0.98) | 0.0002 | 28 | 0.96 (0.94 -0.98) | 0.00004 |  |
|  |  | Egger |  | 0.95 (0.92-0.98) | 0.002 |  | 0.95 (0.93 -0.97) | 0.0003 |  |
|  |  | Weighted Median |  | 0.94 (0.92-0.97) | 0.00001 |  | 0.95 (0.94 -0.97) | 0.0000005 |  |
|  |  | Weighted Mode |  | 0.94 (0.92-0.97) | 0.0002 |  | 0.96 (0.94 -0.97) | 0.00006 |  |
|  |  |  |  |  |  |  |  |  |  |
|  |  |  |  |  |  |  |  |  |  |
| **DHA** | 40 | IVW |  | 0.95 (0.92-0.98) | 0.001 | 37 | 0.96 (0.94 -0.98) | 0.00019 |  |
|  |  | Egger |  | 0.94 (0.89-0.98) | 0.01 |  | 0.94 (0.91 -0.97) | 0.00048 |  |
|  |  | Weighted Median |  | 0.93(0.89-0.96) | 0.00003 |  | 0.94 (0.92 -0.96) | 0.00000069 |  |
|  |  | Weighted Mode |  | 0.92(0.89-0.96) | 0.0001 |  | 0.94 (0.92 -0.97) | 0.000050 |  |
|  |  |  |  |  |  |  |  |  |  |
|  |  |  |  |  |  |  |  |  |  |
| **EPA** | 39 | IVW |  | 0.92 (0.88 - 0.96) | 0.0002 | 38 | 0.94 (0.91 - 0.97) | 0.0003 |  |
|  |  | Egger |  | 0.91 (0.87 - 0.96) | 0.002 |  | 0.93 (0.89 - 0.96) | 0.0004 |  |
|  |  | Weighted Median |  | 0.91 (0.88 - 0.95) | 0.00001 |  | 0.93 (0.9 - 0.96) | 0.000008 |  |
|  |  | Weighted Mode |  | 0.92 (0.87 - 0.96) | 0.001 |  | 0.94 (0.91 - 0.97) | 0.0002 |  |
|  |  |  |  |  |  |  |  |  |  |
|  |  |  |  |  |  |  |  |  |  |
| **OMEGA 6** |  |  |  |  |  |  |  |  |  |
|  |  |  |  |  |  |  |  |  |  |
| **Omega 6** | 50 | IVW |  | 1.01 (0.97-1.05) | 0.60 | 49 | 1.02 (0.99 -1.04) | 0.24 |  |
|  |  | Egger |  | 1.01 (0.92-1.06) | 0.68 |  | 0.97 (0.92 -1.02) | 0.23 |  |
|  |  | Weighted Median |  | 0.99 (0.94-1.03) | 0.61 |  | 1.00 (0.97 -1.03) | 0.92 |  |
|  |  | Weighted Mode |  | 0.98 (0.93-1.04) | 0.48 |  | 1.00 (0.96 -1.04) | 0.95 |  |

## S7. Reverse MR results

Reverse MR of using genome wide significant SNPs (p<5 x 10^-8^) from the PGC MDD GWAS (n=480,539) and UKBB rMDD cohort (n=80,933) as exposures, and DHA and Total Omega 3 Fatty Acids (n=115,078) as outcomes.

|  |  | Major Depressive Disorder (MDD) | | |  | Recurrent Depression (rMDD) | | |
| --- | --- | --- | --- | --- | --- | --- | --- | --- |
|  |  | nSNP | Result | p |  | nSNP | Result | p |
| Total Omega 3 | Inverse variance weighted | 27 | 1.03 (0.98 -1.09) | 0.23 |  | 3 | 1.01 (0.96-1.07) | 0.89 |
|  | MR Egger |  | 0.89 (0.75 -1.07) | 0.24 |  |  | 0.90 (0.74-1.09) | 0.63 |
|  | Weighted median |  | 1.05 (0.98 -1.13) | 0.19 |  |  | 0.98 (0.91-1.05) | 0.73 |
|  | Weighted mode |  | 1.06 (0.92-1.23) | 0.42 |  |  | 0.97 (0.86-1.10) | 0.75 |
|  |  |  |  |  |  |  |  |  |
| DHA | Inverse variance weighted | 27 | 1.01 (0.74-1.09) | 0.73 |  | 3 | 1.01 (0.96-1.07) | 0.69 |
|  | MR Egger |  | 0.90 (0.74-1.09) | 0.28 |  |  | 0.90 (0.74-1.09) | 0.69 |
|  | Weighted median |  | 0.98 (0.91-1.05) | 0.53 |  |  | 0.98 (0.91-1.05) | 0.42 |
|  | Weighted mode |  | 0.97 (-.86-1.10) | 0.65 |  |  | 0.97 (0.86-1.10) | 0.47 |
|  |  |  |  |  |  |  |  |  |

## S8. Comparison of multivariable MR models using different instruments.

All MVMR models using UKBB instruments were suggestive of heterogeneity (with p value for conditional Q statistics <0.05), due to the number of SNPs identified in the UKBB GWAS studies. Furthermore, in model 3, which used UKBB instruments with CHARGE EPA and DHA effect sizes, our conditional F statistics were <10, risking weak instrument bias, which can bias MVMR estimates in unpredictable directions. We therefore undertook further sensitivity analyses, using genetic instruments derived from prior GWAS studies of omega 3 and 6 fatty acids to check consistency of results and try to overcome heterogeneity. The IEU Open GWAS Database id of the relevant GWAS is included in each table for reference. Three tables are presented below for each of the MVMR models in the main manuscript. For each of the alternate MVMR models, the model presented in the main manuscript is presented first (i.e. 1a, 2a, 3a), with alternative models used as sensitivity analyses below (1b, 2b, 3b etc).

For each MVMR model, conditional F statistics and Q statistics were calculated assuming phenotypic covariances below, which had been estimated using the PhenoSpD R package:

Model 1: Total Omega 3: Total Omega 6= 0.77

Model 2: Total Omega 3: Triglycerides 0.58;

Total Omega 3: HDL -0.0668;

Total Omega 3: LDL 0.49;

HDL: LDL 0.1;

HDL: Triglycerides -0.437;

Triglycerides: LDL 0.222)

Model 3: EPA: DHA 0.46.

### Model 1

In model 1b and c, we extracted instruments from Total Omega 3 and Total Omega 6 GWAS studies by Kettunen et al.^6^ In model 1b, SNP-exposure effects were also extracted from the Total Omega 3 and Total Omega 6 Kettunen GWAS’s, resulting in four SNPs for Total Omega 3, and 12 SNPs for Total Omega 6. As the improvement in heterogeneity was modest, (Q=18, p=0.06), model 1c was also included, which used the Kettunen instruments, with SNP-effect sizes from the more precise estimates in UKBB. In this model the heterogeneity was reduced further (Q=14, p=0.16). MVMR effects of Total Omega 3 remained consistent in all three models, though the adverse effect of Total Omega 6 was attenuated.

Table S8a. Alternative MVMR Model 1: The effect of Total Omega 3 on MDD accounting for Total Omega 6.

The ‘Instrument Source’ column denotes the IEU Open GWAS database id of the GWAS study from which genetic instruments were identified, and ‘Effect sizes’ denotes the GWAS id of the SNP-exposure GWAS. nSNP shows the number of SNPs, OR (95% CI) gives the Odds Ratio for MDD (with 95% Confidence Intervals) and p value (p).

F represents the conditional F statistic for each exposure, and Q (p) gives the conditional heterogeneity (and p value).

| ***Model*** | ***Instrument Source*** | ***Effect sizes*** | ***nSNP*** | ***OR (95% CI)*** | ***p*** | ***F*** | ***Q (p)*** |
| --- | --- | --- | --- | --- | --- | --- | --- |
|  |  |  |  |  |  |  |  |
| ***Exposure*** | ***GWAS id*** | ***GWAS id*** |  |  |  |  |  |
|  |  |  |  |  |  |  |  |
| ***MODEL 1a.*** | UK Biobank | UK Biobank |  |  |  |  |  |
|  |  |  |  |  |  |  |  |
| Total Omega 3 | met-d-Omega_3^19^ | met-d-Omega_3^19^ | 43 | 0.93 (0.90-0.97) | 0.0001 | 268 | 96 (0.01) |
| Total Omega 6 | met-d-Omega_6^19^ | met-d-Omega_6^19^ | 49 | 1.07 (1.02-1.12) | 0.01 | 165 |  |
|  |  |  |  |  |  |  |  |
|  |  |  |  |  |  |  |  |
| ***MODEL 1b.*** | Kettunen | Kettunen |  |  |  |  |  |
|  |  |  |  |  |  |  |  |
| Total Omega 3 | met-c-855^6^ | met-c-855^6^ | 13 | 0.90 (0.84- 0.97) | 0.01 | 28 | 18 (0.06) |
| Total Omega 6 | met-c-856^6^ | met-c-856^6^ | 13 | 1.03 (0.98- 1.09) | 0.21 | 41 |  |
|  |  |  |  |  |  |  |  |
|  |  |  |  |  |  |  |  |
| ***MODEL 1c.*** | Kettunen | UK Biobank |  |  |  |  |  |
|  |  |  |  |  |  |  |  |
| Total Omega 3 | met-c-855^6^ | met-d-Omega_3^19^ | 13 | 0.94 (0.90- 0.98) | 0.002 | 1142 | 14 (0.16) |
| Total Omega 6 | met-c-856^6^ | met-d-Omega_6^19^ | 13 | 1.02 (0.96- 1.08) | 0.6 | 379 |  |
|  |  |  |  |  |  |  |  |

### Model 2

Heterogeneity in model 2 was harder to overcome, due to the number of simultaneous exposures, alongside the quantity of UK Biobank genetic instruments for each. The use of Kettunen GWAS instruments for exposures improved heterogeneity only marginally (Q= 46 (p=0.08); and Q=48 (p=0.06)) when using SNP-effect sizes from Kettunen (i.e. model 2b), and UK Biobank (i.e. model 2c) respectively. We therefore selected Triglycerides as a single simultaneous exposure alongside Total Omega 3 fatty acids. Triglycerides were selected as the strongest of the other lipid exposures in models 2a-2c, and also as highlighted in a previous MR study of lipids on major depression.^19^ The heterogeneity remained high in the MVMR model of Total Omega 3 and Triglycerides using UKBB instruments (i.e. model 2d), but was greatly reduced using Kettunen instruments (Q=11 (p=0.35)) with results consistent among all analyses.

Table S8b. Alternative MVMR Model 2: The effect of Total Omega 3 on MDD accounting for correlated lipids.

The ‘Instrument Source’ column denotes the IEU Open GWAS database id of the GWAS study from which genetic instruments were identified, and ‘Effect sizes’ denotes the GWAS id of the SNP-exposure GWAS. nSNP shows the number of SNPs, OR (95% CI) gives the Odds Ratio for MDD (with 95% Confidence Intervals) and p value (p).

F represents the conditional F statistic for each exposure, and Q (p) gives the conditional heterogeneity (and p value).

| ***Model*** | ***Instruments*** | ***Effect sizes*** | ***nSNP*** | ***OR (95% CI)*** | ***p*** | ***F*** | ***Q (p)*** |
| --- | --- | --- | --- | --- | --- | --- | --- |
|  | ***GWAS id*** | ***GWAS id*** |  |  |  |  |  |
| ***Exposure*** |  |  |  |  |  |  |  |
|  |  |  |  |  |  |  |  |
| ***MODEL 2a*** | **UK Biobank** | **UK Biobank** |  |  |  |  |  |
|  |  |  |  |  |  |  |  |
| Triglycerides | ieu-b-111^12^ | ieu-b-111^12^ | 388 | 1.08 (1.03-1.13) | 0.002 | 30 | 682 (9.88E-18) |
| HDL cholesterol | Ieu-b-109^12^ | Ieu-b-109^12^ | 387 | 1.06 (1.01 -1.10) | 0.01 | 42 |  |
| LDL cholesterol | Ieu-b-110^12^ | Ieu-b-110^12^ | 388 | 0.98 (0.93-1.03) | 0.37 | 43 |  |
| Omega-3 fatty acids | met-d-Omega_3^19^ | met-d-Omega_3^19^ | 387 | 0.96 (0.93 -0.98) | 0.001 | 25 |  |
|  |  |  |  |  |  |  |  |
|  |  |  |  |  |  |  |  |
| ***MODEL 2b*** | **Kettunen** | **Kettunen** |  |  |  |  |  |
|  |  |  |  |  |  |  |  |
| Triglycerides | met-c-934^6^ | met-c-934^6^ | 39 | 1.10 (1.03 - 1.17) | 0.01 | 10 | 46 (0.08) |
| HDL cholesterol | met-c-864^6^ | met-c-864^6^ | 39 | 1.02 (0.97 - 1.07) | 0.39 | 11 |  |
| LDL cholesterol | met-c-895^6^ | met-c-895^6^ | 39 | 0.98 (0.95 - 1.01) | 0.21 | 21 |  |
| Omega-3 fatty acids | met-c-855^6^ | met-c-855^6^ | 39 | 0.91 (0.85 - 0.98) | 0.02 | 8 |  |
|  |  |  |  |  |  |  |  |
|  |  |  |  |  |  |  |  |
| ***MODEL 2c*** | **Kettunen** | **UK Biobank** |  |  |  |  |  |
|  |  |  |  |  |  |  |  |
| Triglycerides | met-c-934^6^ | ieu-b-111^12^ | 39 | 1.06 (1.00 - 1.11) | 0.03 | 244 | 48 (0.06) |
| HDL cholesterol | met-c-864^6^ | Ieu-b-109^12^ | 39 | 1.01 (0.96 - 1.06) | 0.63 | 193 |  |
| LDL cholesterol | met-c-895^6^ | Ieu-b-110^12^ | 39 | 1.00 (0.96 - 1.04) | 0.90 | 218 |  |
| Omega-3 fatty acids | met-c-855^6^ | met-d-Omega_3^19^ | 39 | 0.95 (0.92 - 0.99) | 0.01 | 168 |  |
|  |  |  |  |  |  |  |  |
|  |  |  |  |  |  |  |  |
| ***MODEL 2d*** | **UK Biobank** | **UK Biobank** |  |  |  |  |  |
|  |  |  |  |  |  |  |  |
| Triglycerides | ieu-b-111^12^ | ieu-b-111^12^ | 279 | 1.04 (1.00 - 1.07) | 0.04 | 103 | 472 (0) |
| Omega-3 fatty acids | met-d-Omega_3 | met-d-Omega_3^19^ | 279 | 0.95 (0.92 - 0.98) | 0.001 | 46 |  |
|  |  |  |  |  |  |  |  |
|  |  |  |  |  |  |  |  |
| ***MODEL 2e*** | **Kettunen** | **Kettunen** |  |  |  |  |  |
|  |  |  |  |  |  |  |  |
| Triglycerides | met-c-934^6^ | met-c-934^6^ | 13 | 1.08 (1.03 - 1.13) | 0.002 | 41 | 11 (0.35) |
| Omega-3 fatty acids | met-c-855^6^ | met-c-855^6^ | 13 | 0.89 (0.84 - 0.94) | 0.0001 | 27 |  |
|  |  |  |  |  |  |  |  |

### Model 3

For MVMR model 3b, MVMF instruments were identified from the Kettunen et al Total Omega 3 GWAS.^6^ For model 3c, the UK Biobank instruments were refined to twelve SNPs that were nominally associated with EPA or DHA long chain fatty acids in the CHARGE consortium (i.e. taken as p<0.01 in either EPA or DHA GWAS). In each model 3 analyses the SNP-exposure effects were extracted from the relevant CHARGE GWAS. Conditional F statistics and heterogeneity were improved in MVMR models 3b and 3c.

We used a pairwise t-test to calculate the likelihood that the difference between the observed effect sizes for each long chain fatty acid exposure in the model had occurred by chance. A t-test for differences between EPA and DHA effect sizes in model 3a and 3b found no evidence that EPA and DHA had differential effects on MDD (p=0.16, and p=0.41), but Model 3c suggested a stronger effect for EPA that DHA (p=0.03).

Table S8c. Alternative MVMR Model 3: The effect of long chain fatty acids EPA and DHA on MDD accounting for each other. As before, the ‘Instrument Source’ column denotes the IEU Open GWAS database id of the GWAS study from which genetic instruments were identified, and ‘Effect sizes’ denotes the GWAS id of the SNP-exposure GWAS. nSNP shows the number of SNPs, OR (95% CI) gives the Odds Ratio for MDD (with 95% Confidence Intervals) and p value (p). F represents the conditional F statistic for each exposure, and Q (p) gives the conditional heterogeneity (and p value). For model 3b, * represents that the instruments were identified from UK Biobank GWAS studies, but refined to include only those associated with CHARGE EPA or DHA p value <0.01.

|  | **Instruments** | **Effect sizes** | **nSNP** | **OR (95% CI)** | **p** | **F** | **Q (p)** | **t-test (p)** |
| --- | --- | --- | --- | --- | --- | --- | --- | --- |
|  | **GWAS id** | **GWAS id** |  |  |  |  |  |  |
|  |  |  |  |  |  |  |  |  |
| ***MODEL 3a*** | **UK Biobank** | **CHARGE** |  |  |  |  |  |  |
|  |  |  |  |  |  |  |  |  |
| EPA | met-d-Omega_3 | Lemaitre | 40 | 0.93 (0.88-0.97) | 0.002 | 9 | 52 (0.05) | 0.16 |
| DHA | met-d-Omega_3 | Lemaitre | 40 | 0.98 (0.92-1.04) | 0.46 | 6 |  |  |
|  |  |  |  |  |  |  |  |  |
|  |  |  |  |  |  |  |  |  |
| ***MODEL 3b*** | **Kettunen** | **CHARGE** |  |  |  |  |  |  |
|  |  |  |  |  |  |  |  |  |
| EPA | met-c-855 | Lemaitre | 5 | 0.91 (0.85 - 0.98) | 0.01 | 38 | 4 (0.16) | 0.41 |
| DHA | met-c-855 | Lemaitre | 5 | 0.98 (0.85 - 1.13) | 0.76 | 14 |  |  |
|  |  |  |  |  |  |  |  |  |
|  |  |  |  |  |  |  |  |  |
| ***MODEL 3b*** | **UKBB** | **CHARGE** |  |  |  |  |  |  |
|  |  |  |  |  |  |  |  |  |
| EPA | met-d-Omega_3* | Lemaitre | 12 | 0.91 (0.87 - 0.95) | 7.14E-06 | 24 | 7 (0.59) | 0.03 |
| DHA | met-d-Omega_3* | Lemaitre | 12 | 0.98 (0.93 - 1.03) | 0.50 | 14 |  |  |
|  |  |  |  |  |  |  |  |  |

Fig S8d. MVMR scatter plots of model 3, showing SNP effects on EPA (accounting for DHA) and DHA (accounting for EPA) on MDD using different instrument sets.

#### UK Biobank (nSNPs = 40)


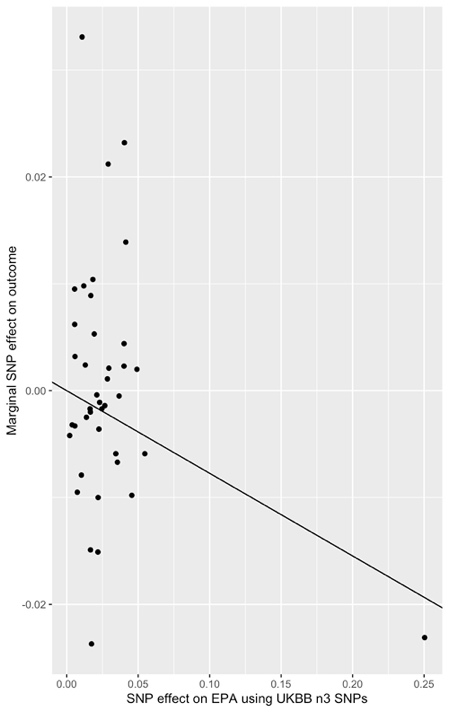

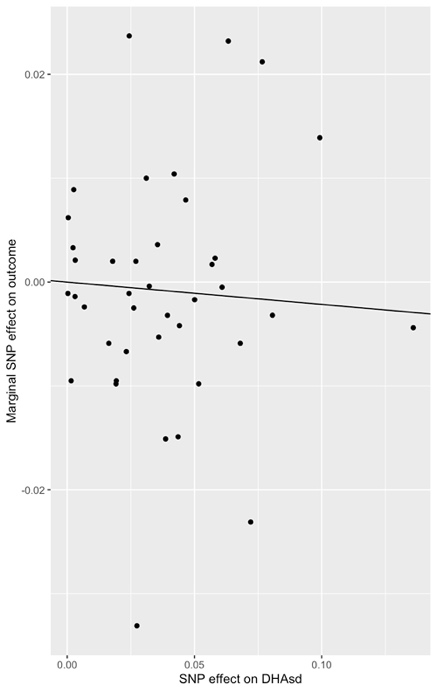


#### Kettunen (nSNPs=5)


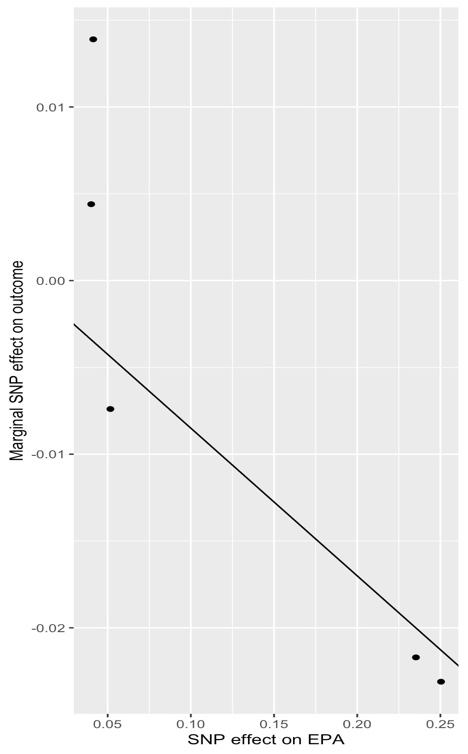

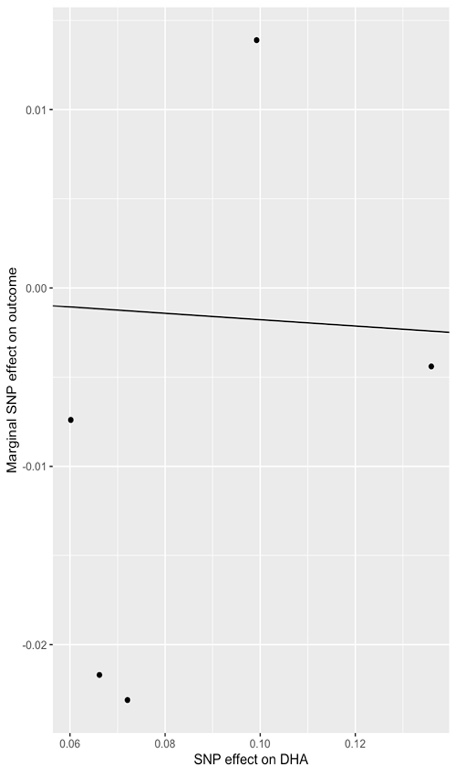


#### UK Biobank SNPs replicated in CHARGE EPA or DHA GWAS (i.e. p<0.01, nSNPs =12)


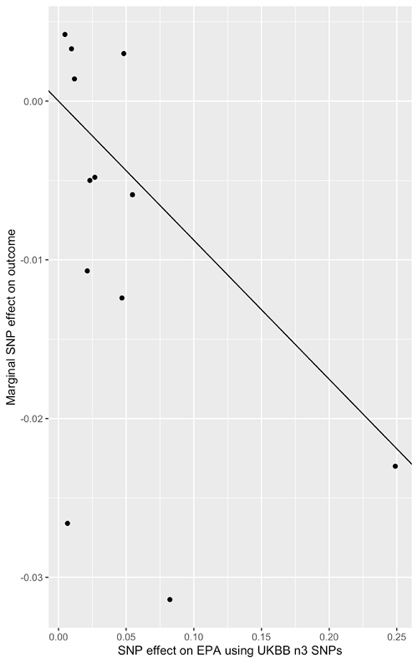

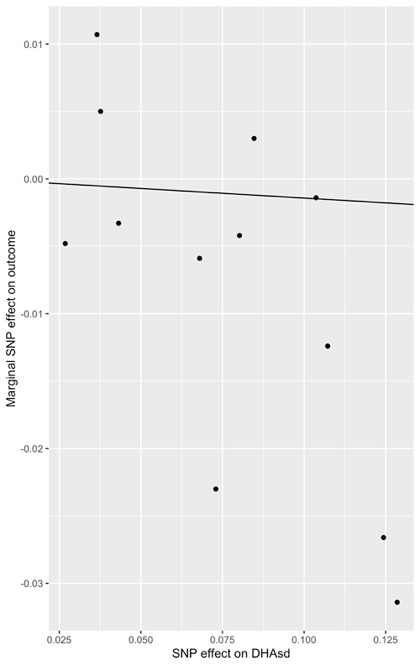


## S9. Colocalization

Results for the colocalization analysis are shown below, using a 500,000 base pair region around *FADS2* (Ensemble reference ENSG00000134824). The posterior probability of association is given in columns H0 to H4.

Analyses using the primary outcome sample (MDD without UKBB, n=480,359), suggested a high posterior probability for a shared causal variant between MDD and total omega 3 (88.9%), DHA (88.9%) and EPA (97.1%). This variant localised to the *FADS2* gene (‘*rs174564’*), strengthening evidence for the association. However, colocalization analyses using the complete MDD (including UKBB participants, n=807,553), and rMDD (n=80,933) samples were less consistent with the hypothesis of a shared causal variant (see table S8a). For rMDD, the high probability of H1 (ie a causal variant for omega 3 only) suggests that this discrepancy is due to low power, in keeping with the smaller sample size. Reasons for the discrepancies between MDD samples with and without UKBB are less clear, with ‘H3’ (i.e. the traits are related but have distinct causal variants) the most likely in the larger sample. As the colocalization plots of these traits appear very similar (figure 2,) we used the gassocplot R package for a more detailed view of the region (Figure S8c). These plots highlight a further variant (*rs198457*) in the region, located on the *DAGLA* gene. Excluding the variant in sensitivity analyses yielded similar results to colocalization using the primary outcome. As the SNP is moderate LD with the *FADS2* variant (r^2^ 0.1), it remains possible that our findings are driven by Linkage Disequilibrium. However, it could represent an additional independent locus in the region, which would violate coloc’s underlying assumption of a single common causal variant.

Table S9a. Probability for a single shared causal variant between traits as given by the ‘coloc’ R package.

| Exposure | Outcome Sample | N | SNPs | H0 (%) | H1 (%) | H2 (%) | H3 (%) | H4 (%) |
| --- | --- | --- | --- | --- | --- | --- | --- | --- |
| Omega 3 | No UKBB | 480,359 | 2,051 | 0.0% | 2.5% | 0.0% | 8.6% | 88.9% |
|  | UKBB included | 807,553 | 2,051 | 0.0% | 0.1% | 0.0% | 72.5% | 27.4% |
|  | rMDD | 80,933 | 2,051 | 0.0% | 91.9% | 0.0% | 4.8% | 3.4% |
|  | UKBB included (minus DAGLA) | 807,553 | 2,050 | 0.0% | 2.5% | 0.0% | 8.6% | 88.9% |
|  |  |  |  |  |  |  |  |  |
| DHA | No UKBB | 480,359 | 2,051 | 0.0% | 2.5% | 0.0% | 8.6% | 88.9% |
|  | UKBB included | 807,553 | 2,051 | 0.0% | 0.1% | 0.0% | 72.5% | 27.4% |
|  | rMDD | 80,933 | 2,051 | 0.0% | 91.9% | 0.0% | 4.8% | 3.4% |
|  | UKBB included (minus DAGLA SNP) | 807,553 | 2,050 | 0.0% | 0.2% | 0.0% | 5.6% | 94.3% |
|  |  |  |  |  |  |  |  |  |
| EPA | No UKBB | 480,359 | 2,051 | 0.0% | 0.7% | 0.0% | 2.2% | 97.1% |
|  | UKBB included | 807,553 | 2,051 | 0.0% | 0.1% | 0.0% | 83.6% | 16.3% |
|  | rMDD | 80,933 | 2,051 | 0.0% | 92.8% | 0.0% | 4.8% | 2.4% |
|  | UKBB included (minus DAGLA SNP) | 807,553 | 2,050 | 0.0% | 0.7% | 0.0% | 2.2% | 97.1% |
|  |  |  |  |  |  |  |  |  |

Figure S9b. Gassocplots to compare MDD (with and without UKBB) and Omega 3. MDD807 is the complete sample including UKBB (n=807,553), MDD is the sample with UKBB removed (n=480,539).
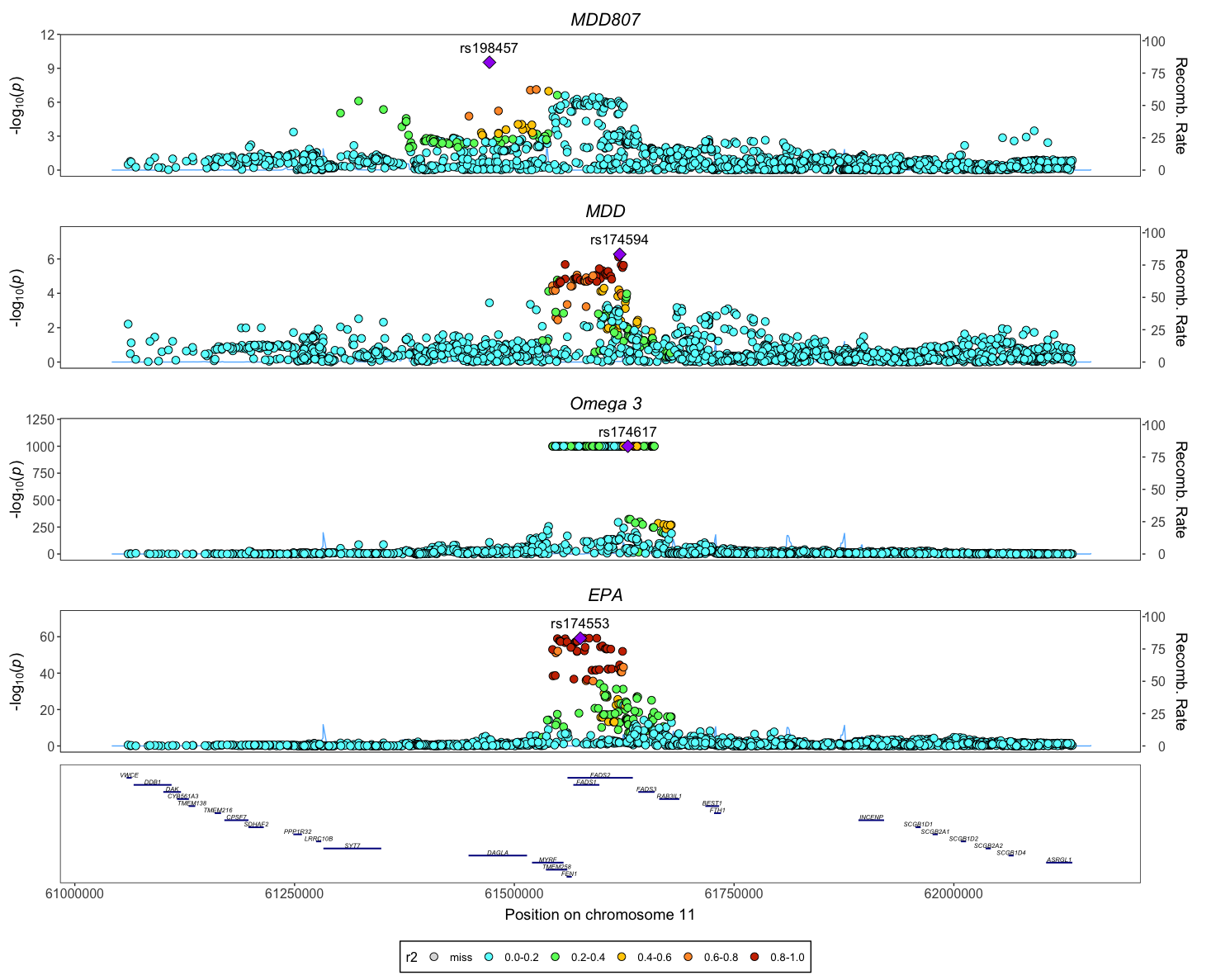


## S10. Phewas results

Complete output from the PheWAS is provided in an excel spreadsheet (“S10 Phewas results.xlsx”). A plot of the top results by category is shown below. Several psychiatrically relevant phenotypes associated with *rs174564* included bipolar disorder (p=8.35E-7, n=51,710), intelligence (p=6.2E-6, n=269,867), cognitive performance (p=5.27E-6, n=257,841), irritability (p=1.8E-7, n=51,710), sleep duration (p=6.3E-9, n=460,099), and daytime naps (p=1.11E-10, n= 337,074).


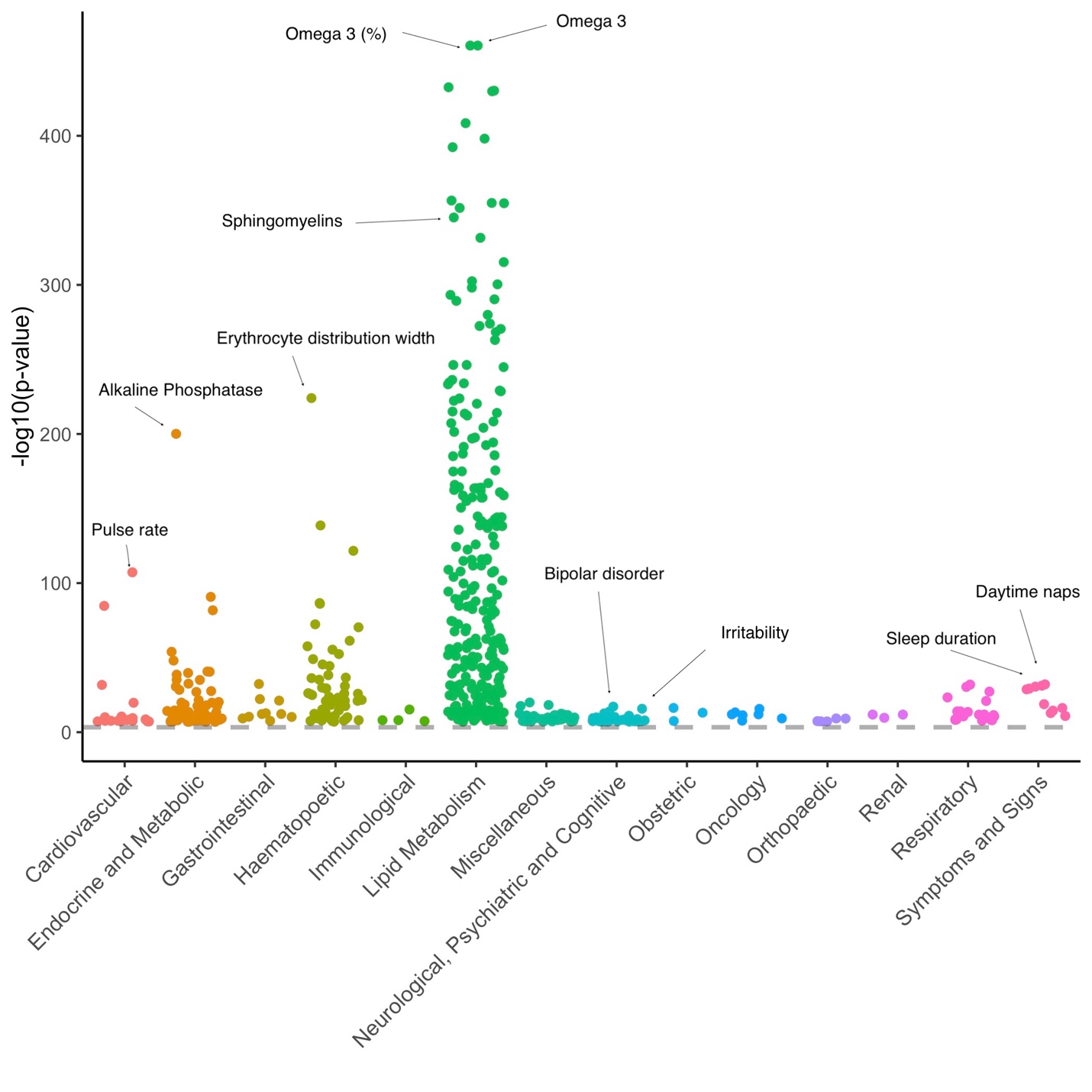


## Supplement References

1. Pierce BL, Ahsan H, Vanderweele TJ. Power and instrument strength requirements for Mendelian randomization studies using multiple genetic variants. Int J Epidemiol. 2011;40(3):740-52.

2. Bowden J, Del Greco MF, Minelli C, Davey Smith G, Sheehan NA, Thompson JR. Assessing the suitability of summary data for two-sample Mendelian randomization analyses using MR-Egger regression: the role of the I^2^ statistic. Int J Epidemiol. 2016;45(6):1961-74.

3. Sanderson E, Davey Smith G, Windmeijer F, Bowden J. An examination of multivariable Mendelian randomization in the single-sample and two-sample summary data settings. Int J Epidemiol. 2019;48(3):713-27.

4. Sanderson E, Windmeijer F. A weak instrument F-test in linear IV models with multiple endogenous variables. J Econometrics. 2016;190(2):212-21.

5. Brion MJA, Shakhbazov K, Visscher PM. Calculating statistical power in Mendelian randomization studies. Int J Epidemiol. 2013;42(5):1497-501.

6. Kettunen J, Demirkan A, Wurtz P, Draisma HH, Haller T, Rawal R, et al. Genome-wide study for circulating metabolites identifies 62 loci and reveals novel systemic effects of LPA. Nat Commun. 2016;7:11122.

7. Nieuwboer HA, Pool R, Dolan CV, Boomsma DI, Nivard MG. GWIS: genome-wide inferred statistics for functions of multiple phenotypes. Am J Hum Genet. 2016;99(4):917-27.

8. Zheng J, Baird D, Borges MC, Bowden J, Hemani G, Haycock P, et al. Recent Developments in Mendelian Randomization Studies. Curr Epidemiol Rep. 2017;4(4):330-45.

9. Hemani G, Tilling K, Davey Smith G. Orienting the causal relationship between imprecisely measured traits using GWAS summary data. Plos Genet. 2017;13(11):e1007081.

10. Burgess S, Freitag DF, Khan H, Gorman DN, Thompson SG. Using Multivariable Mendelian Randomization to Disentangle the Causal Effects of Lipid Fractions. Plos One. 2014;9(10).

11. Sanderson E, Spiller W, Bowden J. Testing and correcting for weak and pleiotropic instruments in two-sample multivariable Mendelian randomization. Stat Med. 2021;40(25):5434-52.

12. Richardson TG, Sanderson E, Palmer TM, Ala-Korpela M, Ference BA, Davey Smith G, et al. Evaluating the relationship between circulating lipoprotein lipids and apolipoproteins with risk of coronary heart disease: A multivariable Mendelian randomisation analysis. Plos Med. 2020;17(3):e1003062.

13. Lemaitre RN, Tanaka T, Tang W, Manichaikul A, Foy M, Kabagambe EK, et al. Genetic loci associated with plasma phospholipid n-3 fatty acids: a meta-analysis of genome-wide association studies from the CHARGE Consortium. Plos Genet. 2011;7(7):e1002193.

14. Wallace C. Eliciting priors and relaxing the single causal variant assumption in colocalisation analyses. Plos Genet. 2020;16(4):e1008720.

15. Wallace C. A more accurate method for colocalisation analysis allowing for multiple causal variants. Plos Genet. 2021;17(9):e1009440.

16. Elsworth B, Lyon M, Alexander T, Liu Y, Matthews P, Hallett J, et al. The MRC IEU OpenGWAS data infrastructure. bioRxiv. 2020:2020.08.10.244293.

17. Borges MC, Haycock PC, Zheng J, Hemani G, Holmes MV, Davey Smith G, et al. Role of circulating polyunsaturated fatty acids on cardiovascular diseases risk: analysis using Mendelian randomization and fatty acid genetic association data from over 114,000 UK Biobank participants. Bmc Med. 2022;20(1):210.

18. Guan WH, Steffen BT, Lemaitre RN, Wu JHY, Tanaka T, Manichaikul A, et al. Genome-Wide Association Study of Plasma N6 Polyunsaturated Fatty Acids Within the Cohorts for Heart and Aging Research in Genomic Epidemiology Consortium. Circ-Cardiovasc Gene. 2014;7(3):321-31.

19. Khandaker GM, Zuber V, Rees JMB, Carvalho L, Mason AM, Foley CN, et al. Shared mechanisms between coronary heart disease and depression: findings from a large UK general population-based cohort. Mol Psychiatr. 2019;19:19.
